# The marine fishes of St Eustatius Island, northeastern Caribbean: an annotated, photographic catalog

**DOI:** 10.3897/zookeys.1007.58515

**Published:** 2020-12-30

**Authors:** David Ross Robertson, Carlos J. Estapé, Allison M. Estapé, Ernesto Peña, Luke Tornabene, Carole C. Baldwin

**Affiliations:** 1 Smithsonian Tropical Research Institute, Balboa, Panama Smithsonian Tropical Research Institute Balboa Panama; 2 150 Nautilus Drive, Islamorada, Florida, 33036, USA Unaffiliated Islamorada United States of America; 3 School of Aquatic and Fishery Sciences and the Burke Museum of Natural History and Culture, University of Washington, Seattle, WA 98107, USA University of Washington Seattle United States of America; 4 Department of Vertebrate Zoology, National Museum of Natural History, Smithsonian Institution, Washington, DC 20560, USA National Museum of Natural History, Smithsonian Institution Washington United States of America

**Keywords:** biodiversity, checklist, faunal completeness, faunal structure, reef-associated bony fishes, SCUBA surveys, submersible surveys

## Abstract

Sint Eustatius (Statia) is a 21 km^2^ island situated in the northeastern Caribbean Sea. The most recent published sources of information on that island’s marine fish fauna is in two non-governmental organization reports from 2015–17 related to the formation of a marine reserve. The species-list in the 2017 report was based on field research in 2013–15 using SCUBA diving surveys, shallow “baited underwater video surveys” (BRUVs), and data from fishery surveys and scientific collections over the preceding century. That checklist comprised 304 species of shallow (mostly) and deep-water fishes. In 2017 the Smithsonian Deep Reef Observation Project surveyed deep-reef fishes at Statia using the crewed submersible Curasub. That effort recorded 120 species, including 59 new occurrences records. In March-May 2020, two experienced citizen scientists completed 62 SCUBA dives there and recorded 244 shallow species, 40 of them new records for Statia. The 2017–2020 research effort increased the number of species known from the island by 33.6% to 406. Here we present an updated catalog of that marine fish fauna, including voucher photographs of 280 species recorded there in 2017 and 2020. The Statia reef-fish fauna likely is incompletely documented as it has few small, shallow, cryptobenthic species, which are a major component of the regional fauna. A lack of targeted sampling is probably the major factor explaining that deficit, although a limited range of benthic marine habitats may also be contributing.

## Introduction

Sint Eustatius island, known locally as Statia, is a 21 km^2^ island in the northeastern Caribbean, and is one of the Leeward Islands in the Lesser Antilles. Until recently there were very few published accounts relating to the marine-fish fauna of Statia. The most comprehensive are represented by two non-governmental organization (NGO) environmental reports to the Statia government by van Kuijk et al. (2015) and Davies and Piontek (2016, 2017). Those two reports referred to only one older scientific publication, by Metzelaar (1919), relating to the fish fauna of that island, among other islands of the Dutch Caribbean. Davies and Piontek (2017) combined their own results from visual surveys with information from BRUV (Baited Remote Underwater Video) surveys by van Kuijk et al. (2015), and a variety of historical scientific collections and fisheries surveys to produce a general list of 307 species (modified to 304, see below), which included both deep- and shallow-water species. In this paper we use the results of deep-reef research using a crewed submersible in 2017 and shallow SCUBA surveys in 2020 to add to the checklist of the island’s marine fish fauna. We also include voucher photographs of most of the species observed and collected during those two surveys. In addition to representing vouchers for the species records, the photographs are intended for use by managers, citizen scientists, recreational divers and fishers who want to identify fishes they see and catch at Statia. Hopefully they will also stimulate future documentation of previously unreported species there. Finally, we compare aspects of the ecological structure of the Statia fauna to that of the regional, Greater Caribbean fauna to assess how complete the faunal inventory is for Statia.

## Materials and methods

### Study area

**Figure 1.**
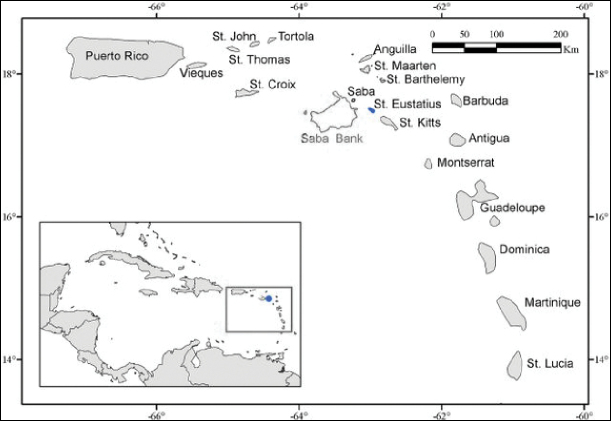
Location of Sint Eustatius. The Caribbean Sea, with the location of Sint Eustatius island indicated in the inset. Source: Hoetjes and Carpenter (2010: fig. 1).

**Figure 2.**
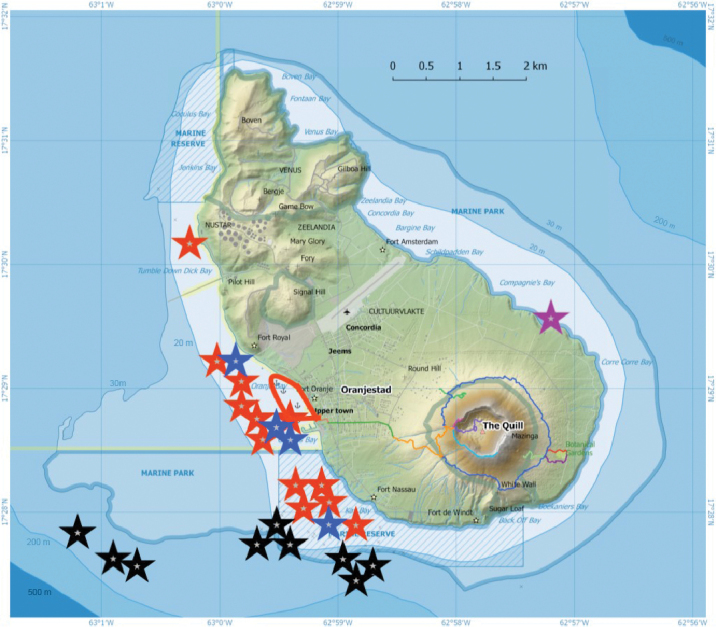
Study sites at Sint Eustatius Island. Location of dive sites during 2017 and 2020: Black stars indicate submersible dives, blue stars 2017 SCUBA dives, red stars 2020 SCUBA dives (some individual stars indicate multiple dives in very close proximity), purple star an intertidal snorkeling site, and the red outline shows limits of the shore-diving area in 2020. See Suppl. material 2: Table S1 for georeferenced date on dive sites. Generalized 20 m, 30 m, 200 m and 500 m isobaths in blue; other lines indicate marine and terrestrial reserve areas. (Base map from Statiaparks, openstreemap.org, CC-BY-SA 2.0 with bathymetry data corrected from CARMABI/WWF/E.Imms (https://www.dcbd.nl/document/bathymetry-map-seas-surrounding-st-eustatius-saba-and-st-maarten, accessed 10 July 2020)

As one of the Dutch Caribbean islands, Statia sits among Saba, Sint Marten and St Kitts and Nevis (Figure 1) and shares a 200-m insular shelf with the last two islands (Suppl. material 1: Figure S1). Statia is surrounded by a narrow 200-m shelf, which is most extensive on the leeward, western side (Figure 2). The island has a limited diversity of marine habitats. It lacks large, deep embayments, particularly on the western side, that would otherwise provide sheltered locations for development of fringing and back-reef areas. Statia has little well-developed coral reef and most reef areas are of relatively low relief. Due to the general degree of exposure of the entire island to ocean swells it lacks any mangroves and has little in the way of seagrass beds, which are now dominated by a non-native species of *Halodule* (van Kuijk et al. 2015; Hoeksema 2016).

The Caribbean Sea, with the location of Sint Eustatius island indicated in the inset. Source: Hoetjes and Carpenter (2010: fig. 1).

## Data sources

### Published species lists

A comprehensive set of species records came from two NGO studies, which were included in a report by Hoeksema (2016). van Kuijk et al. (2015) recorded 107 species during “baited underwater video surveys” (BRUVs) at 104 sites in shallow water (<30 m deep) scattered around all sides of the island in 2013. Davies and Piontek (2016, 2017) recorded 206 species during 38 of their own shallow, roving-diver surveys in 2015, and augmented that list with a list of species they extracted from historical literature, museum records (from major online aggregators, see below), photographs of fishes caught at the island that they obtained from various sources, and fisheries surveys. They added the species recorded by van Kuijk et al. (2015) to those they had seen and extracted from other sources to produce a combined list of 307 species.

### Research in 2017 and 2020

In 2017 the Smithsonian Institution’s Deep Reef Observation Project (DROP) worked with the crewed submersible Curasub to make collections and observations on deep-reef fishes at Statia, to complement similar prior work at the Antillean islands of Dominica and Curaçao (e.g., Baldwin et al. 2018). The submersible was launched close to shore from the tender vessel R/V Chapman and towed by a surface boat to locations along the outer reef slope off the southwest coast where the shallow reef flat transitioned to the slope (~ 40–50 m). Eleven submersible dives were made off the southwestern edge of the island’s 200 m platform (see Figure 2, and Suppl. material 2: Table S1). Each dive lasted approximately five hours and reached a maximum depth of 143–305 m, depending on the habitat at that particular site. Submersible surveys follow the methods used by Baldwin et al. (2018). Dives were roving surveys with the submersible facing the reef and moving laterally while slowly descending the slope. Periodically, stops were made to collect specimens using an anesthetic (quinaldine in ethanol) ejection system attached to the sub’s manipulator arms, coupled with a suction pump attached to one arm that emptied into a holding chamber. On five of the eleven dives visual records of fishes were obtained by CB and LT, who were seated in the front of the submersible and linked their sightings of identifiable fishes to depth measurements recorded from a digital depth gauge inside the submersible. High-definition video was also recorded on five dives from a camera mounted on the front of the sub. Five scuba-based collection dives to a maximum depth of 20 m were also made by LT and CB, who were targeting sponge-associated gobies. A total of 210 specimens was collected, and 6475 individuals were recorded from visual observations during the SCUBA and submersible dives by DROP. Some of those specimens represent undescribed species or belong to groups with uncertain taxonomy.

Two of the authors, CJE and AME, are citizen scientists with extensive experience photographing reef fishes at various sites in the Greater Caribbean. In 2020 they spent two months (mid-March to mid-May) living at Statia and SCUBA diving daily to obtain photographic vouchers of the fishes they observed. They made 62 dives, each of approximately one-hour duration, at depths between 1–30 m on both hard-reef, sand, rubble and seagrass habitats, as well as on sunken wrecked ships. Half of those dives were nearshore in a restricted area, as, during the second half of their stay at the island, they lacked dive-boat support and were able to dive only from the shoreline (see Figure 2, and Suppl. material 2: Table S1). During those dives CJE and AME accumulated photographs of the great majority of fish species they saw. They also obtained recent photographs of a few species taken by local divers and fishers at Statia that they did not see or photograph themselves.

### Online aggregators

In addition, we also assessed information provided by three major aggregators of online georeferenced location data on marine fishes (GBIF https://www.gbif.org/, OBIS https://obis.org/, and FishNet2 http://www.fishnet2.net/search.aspx, all accessed on 7 May 2020), searching for records in ~ 120-km^2^ quadrat based on Admiralty Chart 487G that encompassed Statia and the surrounding shelf area: the area bounded by 17.433°N to 17.533°N and – 62.933°W to – 63.033°W. That quadrat contained almost 100 km^2^ of marine habitat. That area is a little larger than and centered on the area shown in Figure 2. Those sites regularly update the information they contain and might have had additional records to those found by Davies and Piontek (2017).

Location of dive sites during 2017 and 2020: Black stars indicate submersible dives, blue stars 2017 SCUBA dives, red stars 2020 SCUBA dives (some individual stars indicate multiple dives in very close proximity), purple star an intertidal snorkeling site, and the red outline shows limits of the shore-diving area in 2020. See Suppl. material 2: Table S1 for georeferenced date on dive sites. Generalized 20 m, 30 m, 200 m and 500 m isobaths in blue; other lines indicate marine and terrestrial reserve areas. (Base map from Statiaparks, openstreemap.org, CC-BY-SA 2.0 with bathymetry data corrected from CARMABI/WWF/E.Imms (https://www.dcbd.nl/document/bathymetry-map-seas-surrounding-st-eustatius-saba-and-st-maarten, accessed 10 July 2020)

## The structure of the Statia reef-fish fauna

### Zoogeography

Members of the entire Statia fauna as currently known (Table 1; hereafter Statia20) were assessed in terms of their global and local geographical ranges, as follows: (a) Endemism – we noted whether each is a Greater Caribbean endemic, or is distributed more widely in the tropical western Atlantic (i.e., to the north and south of the Greater Caribbean, or on both sides of the Atlantic, or in the Indo-Pacific as well as the Atlantic). (b) Geographical range size – we noted which species have small geographical ranges within the Greater Caribbean, which we defined as ranges that span no more than one third of the area of that region (based on maps of their ranges in Robertson and Van Tassell 2019).

### Ecological structure

The research during 2017–2020 was aimed at documenting the reef-associated bony fishes of Statia. For analyses of the structure of the Statia20 fauna we assigned those species to the following ecological groups (following Robertson and Tornabene 2020): Reef-associated fishes include demersal and benthic species that use hard substrata (coral- and rock reefs), and soft bottoms (sand, gravel, mud, seagrass and macroalgal beds growing on sediment, estuaries and mangroves) immediately adjacent to or within the matrices of reefs. Benthic species are restricted to living on and in the bottom, while demersal species use both the bottom and the near-bottom water column. Cryptobenthic fishes are visually and/or behaviorally cryptic due to their form and coloration, and to their maintaining a close association with the benthos, directly on or within it. Small size (here maximum total length (TL) ≤10 cm) also is thought to be important for crypsis among such species. Core families of cryptobenthic reef fishes (Core CRFs) (see Brandl et al. 2018, 2019) found in the western Atlantic include the Apogonidae, Blenniidae, Bythitidae, Callionymidae, Chaenopsidae, Dactyloscopidae, Gobiesocidae, Gobiidae, Grammatidae, Labrisomidae, Opistognathidae, Syngnathidae, Tripterygiidae. To these families we added the Dinematichthyidae, which was split from the Bythitidae by Møller et al. (2016) shortly before Brandl et al. (2018) assembled their list of Core CRF families, and contains many shallow, reef-associated species. Species in the list are divided into two depth classes, based on their depth ranges: shallow species are those commonly found above 40 m depth, and deep species are those entirely or largely restricted to depths below 40 m.

In the Greater Caribbean region reef-associated bony fishes comprise ~ 900 species from 304 genera in 76 families (Robertson and Tornabene 2020). Reef-fish faunas of deep reefs down to ~ 250 m are dominated by the same set of families that are common on shallow reefs (Baldwin et al. 2018). At the regional level ~ 95% of those reef-associated species are non-pelagic, demersal and benthic forms, which were the focus of the 2017–2020 research at Statia. The relative abundance of the different ecological groups in the Statia20 fauna was compared to: (a) that of the regional fauna to assess similarities and differences; (b) that of the Statia fauna of Davies and Piontek (2017) (hereafter Statia17) to assess any changes; and (c) that of the Saba Exclusive Economic Zone (EEZ) (which includes Statia) (hereafter Saba17) prior to the 2017–2020 research to assess the identity and ecotypes of species that, although they are not on the Statia20 list, do occur very near Statia. Finally, we compare the relative abundances of the different ecogroups in the Statia20 fauna to those at one of the best sampled reefs in the Greater Caribbean, which has the largest published fauna: Alligator Reef in the Florida Keys (see Williams et al. 2010). The Alligator reef faunal checklist was recently updated and expanded (Starck et al. 2017; Estapé et al. 2020; hereafter Alligator20), and, hence, should provide a useful comparison.

A list of reef-associated fishes known from Alligator Reef was extracted from the list in Starck et al. (2017), and Estapé et al (2020) by comparing it to the checklist of regional reef-associated fishes of Robertson and Tornabene (2020). A faunal list for the Saba EEZ (see Suppl. material 1: Figure S1) was obtained by using the “Species List Assembly” tool in Robertson and Van Tassell (2019) (https://biogeodb.stri.si.edu/caribbean/en/research/index/list), as follows: within the tool the following combination of factors was selected – all species/ political area/ Saba EEZ. The confirmed species on the list generated (those with actual records within that EEZ) were then used here. A few species represented solely by data from the 2017–20 research at Statia that were on the Saba EEZ list generated by that tool were excluded from that list for the present comparisons.

## Results

### Modifications to the list of Davies and Piontek (2017)

We reduced the number of species on the list of Davies and Piontek (2017) (which is unchanged from that of Davies and Piontek 2016) from 307 to 304 through three deletions. Those included *Emblemariopsisoccidentalis* Stephens, 1970, *Pteroismiles* (Bennett, 1828) and *Enneanectespectoralis* (Evermann & Marsh, 1899). Those authors recorded *E.occidentalis* and provided a photograph (on p 75 of Davies and Piontek 2016) of the fish they gave this name. However, *E.occidentalis* is now known to be restricted to the Bahamas (B Victor pers. comm., 26 May 2020). Authors CJE and AME photographed two species of this genus at Statia, *E.bahamensis* and *E.carib*. While *E.carib* (and *E.occidentalis*) has a simple ocular cirrus, *E.bahamensis* lacks such a cirrus. As the fish in Davies and Piontek’s (2016) photograph clearly has an ocular cirrus it cannot be *E.bahamensis*. B Victor (pers. comm., 26 May 2020) examined that photograph and concluded it is of either *E.carib* or possibly *E leptocirris* Stephens 1970, which has an ocular cirrus and is known from the Puerto Rican plateau, 185 km from Statia. Hence, we deleted *E.occidentalis* from the list but did not include *E.leptocirris* due to the uncertain identification of that photograph. The Indo-west Pacific lionfish *P.volitans* apparently is a hybrid of two Indo-west Pacific species, and the West Atlantic population of this lionfish appears to be composed almost entirely of *P.volitans* (Wilcox et al. 2018). Hence, we excluded *P.miles* from the list as it is unlikely to be present at Statia and any such an occurrence has not been confirmed genetically. Davies and Piontek (2017) included both *Enneanectespectoralis* and *E.jordani* on the list. However, we excluded *E.pectoralis* as it recently has been shown to be a synonym of *E.jordani* (see Victor 2017). In addition, we changed the names for two of Davies and Piontek’s (2017) species: Davies and Piontek (2017) recorded *L.campechanus* (Poey, 1860), which is now known to be restricted to the Gulf of Mexico and US area. The taxonomic separation of *L.purpureus*, which ranges from the Caribbean to Brazil, from *L.campechanus* was recently confirmed by da Silva et al. (2020). Davies and Piontek (2017) recorded *S.mitsukurii* Jordan & Snyder, 1903. However, the Greater Caribbean population was recently renamed *S.clarkae* (see Ehemann et al. 2019) and *S.mitsukurii* is now regarded as restricted to the Eastern Atlantic and Indo-west Pacific. Those changes reduced the Statia17 list from 307 to 304 species.

### Additions from other sources

The Van Kuijk et al. (2015) list of 106 species contained one species (*Chilomycterusschoe*pfi) not included by Davies and Piontek (2017) in their list. FishNet2 supplies data based on museum records to GBIF and all 34 species records from FishNet2 were also in the GBIF list and are not separately indicated in Table 1. The GBIF list included 103 species, and, after discounting the 27 DROP2017 collection records included therein, none of the 76 remaining species represented “new” records that are not on the Davies and Piontek (2017) list. OBIS, which also supplies data to GBIF, produced 37 records, 13 of which (all common, widely distributed species) were not in the GBIF list, but only one of which (*Coryphaenahippurus*) was not in any other database.

DROP recorded a total of 120 species, 59 of which were not in any other list, except for two new records it shared with the Estapé 2020 list. Eight of those 59 records are of species that have yet to be described and named. The Estapé 2020 list includes 244 records, 40 of them new, plus two other new additions they share with DROP. Summing the deletions and additions from various sources produced a total of 406 species for the Statia20 checklist (see Table 1).

**Table 1.** Updated checklist of marine fishes from Sint Eustatius Island, 2020. Key to column headings and entries: **DROP** – CP = collected and photographed; C collected only; V = visual observation only; **Estapé** – P = photographed by CJE and AME; (P) photographed by 3^rd^ parties; V = visual observation only by CJE and AME. **New** – species is a new record resulting from 2017–20 research, and its source. Other sources of species records are van Kuijk et al. 2015**(vK15)**, Davies and Piontek 2017**(DP17), GBIF**, and **OBIS**. DROP in GBIF indicates record in GBIF is derived from 2017 DROP collection specimens deposited in the fish collection of the US National Museum of Natural History. FishNet 2 records are not indicated separately because all such records are included by GBIF. **NA**- not applicable to non-native *Pteroisvolitans*. **Plate** – number indicates supplemental plate containing the voucher photograph of that species. **Zoogeography (Zoo)**- Global geographic range of species; GC = Greater Caribbean endemic; NWA = GC plus temperate eastern USA; WA = GC plus Brazil; TA = WA plus central or East Atlantic; PAC = Pacific; EP = East Pacific; IWP = Indo-west Pacific; PAN = Pantropical or Circumglobal. **Range** – extent of geographic range – L = range limited, not more than one third of the Greater Caribbean; remainder are more widely distributed in that region. **Deep** – species entirely or largely restricted to depths below 40 m. **Yes** indicates a species conforms to the heading of the column; ? indicates insufficient data.

Species in families	English common name	New	DROP	Estapé	vK15	DP17	GBIF	OBIS	Plate	Zoo	Range	Deep
ACANTHURIDAE												
*Acanthuruschirurgus* (Bloch, 1787)	Doctorfish		V	P	Yes	Yes	Yes	Yes	1	GC		
*Acanthuruscoeruleus* Bloch & Schneider, 1801	Blue Tang		V	P	Yes	Yes	Yes	Yes	1	GC		
*Acanthurustractus* Poey, 1860	Northern Ocean Surgeonfish		V	P	Yes	Yes	Yes	Yes	1	GC		
ACHIRIDAE												
*Gymnachirusnudus* Kaup, 1858	Flabby Sole	Estapé		P					1	GC		
ACROPOMATIDAE												
*Synagropsbellus* (Goode & Bean, 1896)	Blackmouth Bass					Yes				WA		Yes
AETOBATIDAE												
*Aetobatusnarinari* (Euphrasen, 1790)	Spotted Eagle Ray			(P)	Yes	Yes			1	WA		
ANTENNARIIDAE												
*Antennariusmultiocellatus* (Valenciennes, 1837)	Longlure Frogfish			P		Yes			1	WA		
*Histriohistrio* (Linnaeus, 1758)	Sargassumfish			(P)		Yes			1	PAN		
APOGONIDAE												
*Apogonaurolineatus* (Mowbray, 1927)	Bridle Cardinalfish					Yes				GC		
*Apogonmaculatus* (Poey, 1860)	Flamefish			P		Yes			1	GC		
*Apogonpillionatus* Bohlke & Randall, 1968	Broadsaddle Cardinalfish	DROP	V							GC		
*Apogonplanifrons* Longley & Hildebrand, 1940	Pale Cardinalfish	Estapé		P					1	WA		
*Apogonpseudomaculatus* Longley, 1932	Twospot Cardinalfish	DROP	C				DROP			WA		
*Apogonquadrisquamatus* Longley, 1934	Sawcheek Cardinalfish			P		Yes			1	WA		
*Apogontownsendi* (Breder, 1927)	Belted Cardinalfish			P		Yes			1	WA		
*Astrapogonpuncticulatus* (Poey, 1867)	Blackfin Cardinalfish	Estapé		V						WA		
*Astrapogonstellatus* (Cope, 1867)	Conchfish					Yes				WA		
*Paroncheilusaffinis* (Poey, 1875)	Bigtooth Cardinalfish		V	P		Yes			1	TA		
*Phaeoptyxconklini* (Silvester, 1915)	Freckled Cardinalfish	Estapé		P					1	GC		
*Phaeoptyxpigmentaria* (Poey, 1860)	Dusky Cardinalfish					Yes			1	TA		
ARGENTINIDAE												
*Argentina stewarti* Cohen & Atsaides, 1969						Yes				GC		Yes
*Glossanodonpygmaeus* Cohen, 1958	Pygmy Argentine	DROP	CP						1	WA		Yes
ATHERINIDAE												
*Atherinaharringtonensis* Goode, 1877	Reef Silverside					Yes				GC		
*Atherinomorusstipes* (Müller & Troschel, 1848)	Hardhead Silverside	Estapé		P					1	WA		
AULOSTOMIDAE												
*Aulostomusmaculatus* Valenciennes, 1841	Atlantic Trumpetfish			P	Yes	Yes	Yes		1	GC		
BALISTIDAE												
*Balistescapriscus* Gmelin, 1789	Gray Triggerfish			P		Yes			1	TA		
*Balistesvetula* Linnaeus, 1758	Queen Triggerfish			P	Yes	Yes		Yes	1	TA		
*Canthidermissufflamen* (Mitchill, 1815)	Ocean Triggerfish		V			Yes				WA		
*Melichthysniger* (Bloch, 1786)	Black Durgon			P	Yes	Yes	Yes	Yes	1	PAN		
*Xanthichthysringens* (Linnaeus, 1758)	Sargassum Triggerfish	DROP	V							WA		
BELONIDAE												
*Platybeloneargalusargalus* (Lesueur, 1821)	Keeltail Needlefish					Yes				WA		
*Tylosuruscrocodilus* (Péron & Lesueur, 1821)	Houndfish			P		Yes			1	PAN		
BLENNIIDAE												
*Entomacrodusnigricans* Gill, 1859	Pearl Blenny			P		Yes			1	GC		
*Hypleurochiluspseudoaequipinnis* Bath, 1994	Oyster Blenny	Estapé		P					1	WA		
*Hypleurochilusspringeri* Randall, 1966	Orangespotted Blenny	Estapé		P					1	GC		
*Hypsoblenniusexstochilus* Bohlke, 1959	Longhorn Blenny			(P)		Yes			2	GC		
*Ophioblenniusmacclurei* (Silvester, 1915)	Redlip Blenny			P		Yes	Yes		2	GC		
*Parablenniusmarmoreus* (Poey, 1876)	Seaweed Blenny			P		Yes			2	WA		
BOTHIDAE												
*Bothuslunatus* (Linnaeus, 1758)	Peacock Flounder			P	Yes	Yes			2	TA		
*Bothusocellatus* (Agassiz, 1831)	Eyed Flounder			P		Yes			2	WA		
*Chascanopsettalugubris* Alcock, 1894	Pelican Flounder					Yes				TA,IWP		Yes
CALLIONYMIDAE												
*Callionymusbairdi* (Jordan, 1888)	Lancer Dragonet			P		Yes			2	WA		
*Foetorepus* species		DROP	CP						13	WA?	?	Yes
CAPROIDAE												
*Antigoniacapros* Lowe, 1843	Deepbody Boarfish	DROP	V							TA,IWP		Yes
CARANGIDAE												
*Alectisciliaris* (Bloch, 1787)	African Pompano					Yes				PAN		
*Caranxbartholomaei* (Cuvier, 1833)	Yellow Jack			P		Yes			2	TA		
*Caranxcrysos* (Mitchill, 1815)	Blue Runner			P		Yes			2	TA		
*Caranxhippos* (Linnaeus, 1766)	Crevalle Jack					Yes				WA		
*Caranxlatus* Agassiz, 1831	Horse-eye Jack			P	Yes	Yes			2	TA		
*Caranxlugubris* Poey, 1860	Black Jack		V		Yes	Yes				PAN		
*Caranxruber* (Bloch, 1793)	Bar Jack		V	P	Yes	Yes	Yes	Yes	2	WA		
*Decapterusmacarellus* (Cuvier, 1833)	Mackerel Scad			P		Yes			2	PAN		
*Decapteruspunctatus* (Cuvier, 1829)	Round Scad			P		Yes			2	TA		
*Elagatisbipinnulata* (Quoy & Gaimard, 1825)	Rainbow Runner			P		Yes			2	PAN		
*Selarcrumenophthalmus* (Bloch, 1793)	Bigeye Scad			P		Yes			2	PAN		
*Seriolarivoliana* Valenciennes, 1833	Almaco Jack			P	Yes	Yes			2	PAN		
*Trachinotusfalcatus* (Linnaeus, 1758)	Permit			P		Yes			2	WA		
*Trachinotusgoodei* Jordan & Evermann, 1896	Palometa			P		Yes			2	WA		
CARCHARHINIDAE												
*Carcharhinusleucas* (Müller & Henle, 1839)	Bull Shark					Yes				PAN		
*Carcharhinuslimbatus* (Müller & Henle, 1839)	Blacktip Shark				Yes	Yes				PAN		
*Carcharhinusperezii* (Poey, 1876)	Reef Shark			V	Yes	Yes				WA		
*Galeocerdocuvier* (Peron & Lesueur, 1822)	Tiger Shark					Yes				PAN		
*Negaprionbrevirostris* (Poey, 1868)	Lemon Shark			P		Yes			2	TA,EP		
CENTROPHORIDAE												
*Centrophorusgranulosus* (Bloch & Schneider, 1801)	Large Gulper Shark					Yes				TA,IWP		Yes
CHAENOPSIDAE												
*Acanthemblemariaaspera* (Longley, 1927)	Roughhead Blenny			P		Yes			2	GC		
*Acanthemblemariamaria* Bohlke, 1961	Secretary Blenny			P		Yes	Yes		2	GC		
*Acanthemblemariaspinosa* Metzelaar, 1919	Spinyhead Blenny			P		Yes	Yes		2	GC		
*Chaenopsislimbaughi* Robins & Randall, 1965	Yellowface Pikeblenny			P		Yes			2	GC		
*Emblemariapandionis* Evermann & Marsh, 1900	Sailfin Blenny			P	Yes	Yes			2	GC		
*Emblemariavitta* Williams, 2002	Ribbon Blenny	Estapé		(P)					2	GC		
*Emblemariopsisbahamensis* Stephens, 1961	Blackhead Blenny	Estapé		P					3	GC	L	
*Emblemariopsiscarib* Victor, 2010	Carib Blenny	Estapé		P					3	GC	L	
CHAETODONTIDAE												
*Chaetodoncapistratus* Linnaeus, 1758	Foureye Butterflyfish		V	P	Yes	Yes	Yes	Yes	3	GC		
*Chaetodonocellatus* Bloch, 1787	Spotfin Butterflyfish			P	Yes	Yes		Yes	3	WA		
*Chaetodonsedentarius* Poey, 1860	Reef Butterflyfish		V		Yes	Yes		Yes		WA		
*Chaetodonstriatus* Linnaeus, 1758	Banded Butterflyfish		V	P	Yes	Yes	Yes	Yes	3	WA		
*Prognathodesaculeatus* (Poey, 1860)	Longsnout Butterflyfish		C	P	Yes	Yes	Yes	Yes	3	WA		
*Prognathodesguyanensis* (Durand, 1960)	Guyana Butterflyfish	DROP	V							GC		Yes
CHAUNACIDAE												
*Chaunaxsuttkusi* Caruso, 1989	Pale-cavity Gaper					Yes				TA		Yes
CHIMAERIDAE												
*Chimaeracubana* Howell Rivero, 1936	Cuban Chimaera					Yes				GC		Yes
*Hydrolagusalberti* Bigelow & Schroeder, 1951	Gulf Chimaera					Yes				GC		Yes
CHLOPSIDAE												
*Chilorhinussuensonii* Lutken, 1852	Seagrass Eel					Yes				WA		
CHLOROPHTHALMIDAE												
*Chlorophthalmusagassizi* Bonaparte, 1840	Shortnose Greeneye					Yes				TA		Yes
*Parasudistruculenta* (Goode & Bean, 1895)	Longnose Greeneye					Yes				WA		Yes
CIRRHITIDAE												
*Amblycirrhituspinos* (Mowbray, 1927)	Redspotted Hawkfish			P		Yes	Yes		3	WA		
CLUPEIDAE												
*Harengulaclupeola* (Cuvier, 1829)	False Pilchard					Yes				WA		
*Harengulahumeralis* (Cuvier, 1829)	Redear Sardine					Yes				GC		
*Jenkinsialamprotaenia* (Gosse, 1851)	Dwarf Herring					Yes				GC		
*Opisthonemaoglinum* (Lesueur, 1818)	Atlantic Thread Herring					Yes				WA		
*Sardinellaaurita* Valenciennes, 1847	Spanish Sardine					Yes				TA		
CONGRIDAE												
*Ariosomabalearicum* (Delaroche, 1809)	Bandtooth Conger	Estapé		(P)					3	TA		
*Heterocongerlongissimus* Gunther, 1870	Brown Garden Eel			P	Yes	Yes	Yes		3	WA		
*Xenomystaxbidentatus* (Reid, 1940)	Twopatched-teeth Conger					Yes				TA		Yes
CORYPHAENIDAE												
*Coryphaenahippurus* Linnaeus, 1758	Dolphinfish							Yes		PAN		
CRURIRAJIDAE												
*Crurirajarugosa* Bigelow & Schroeder, 1958	Rough Leg Skate					Yes				GC		Yes
CYNOGLOSSIDAE												
*Symphurusmarginatus* (Goode & Bean, 1886)	Margined Tonguefish					Yes				WA		Yes
DACTYLOPTERIDAE												
*Dactylopterusvolitans* (Linnaeus, 1758)	Flying Gurnard			P	Yes	Yes	Yes		3	TA		
DASYATIDAE												
*Hypanusamericanus* Hildebrand & Schroeder, 1928	Southern Stingray			P	Yes	Yes	Yes		3	WA		
DIODONTIDAE												
*Chilomycterusantillarum* Jordan & Rutter, 1897	Web Burrfish			P		Yes	Yes		3	WA		
*Chilomycterusschoepfii* (Walbaum, 1792)	Striped Burrfish				Yes					NWA		
*Diodonholocanthus* Linnaeus, 1758	Balloonfish			P		Yes			3	PAN		
*Diodonhystrix* Linnaeus, 1758	Porcupinefish			P	Yes	Yes	Yes		3	PAN		
DIRETMIDAE												
*Diretmusargenteus* Johnson, 1864	Silver Spinyfish					Yes				PAN		Yes
ECHENEIDAE												
*Echeneisnaucrates* Linnaeus, 1758	Sharksucker			P	Yes	Yes	Yes		3	PAN		
*Echeneisneucratoides* Zuiew, 1786	Whitefin Sharksucker	Estapé		P					3	NWA		
*Remoraremora* (Linnaeus, 1758)	Remora					Yes				PAN		
EPHIPPIDAE												
*Chaetodipterusfaber* (Broussonet, 1782)	Atlantic Spadefish					Yes				WA		
ETMOPTERIDAE												
*Etmopterushillianus* (Poey, 1861)	Caribbean Lantern Shark					Yes				NWA		Yes
*Etmopterusrobinsi* Schofield & Burgess, 1997	West Indian Lantern Shark					Yes				GC		Yes
FISTULARIIDAE												
*Fistulariatabacaria* Linnaeus, 1758	Bluespotted Cornetfish			P	Yes	Yes			3	TA		
GERREIDAE												
*Eucinostomusjonesii* (Gunther, 1879)	Slender Mojarra					Yes				WA		
*Eucinostomuslefroyi* (Goode, 1874)	Mottled Mojarra			P		Yes			3	WA		
*Gerrescinereus* (Walbaum, 1792)	Yellowfin Mojarra					Yes				WA		
GINGLYMOSTOMATIDAE												
*Ginglymostomacirratum* (Bonnaterre, 1788)	Nurse Shark			(P)	Yes	Yes	Yes		3	TA		
GOBIESOCIDAE												
*Derilissuslombardii* Sparks & Gruber, 2012	Tailspot Clingfish	DROP	CP						3	GC		Yes
GOBIIDAE												
*Antilligobiusnikkiae* Van Tassell & Colin, 2012	Sabre Goby	DROP	CP				DROP		3	GC		Yes
*Bathygobiusantilliensis* Tornabene, Baldwin & Pezold, 2010	Antilles Frillfin	Estapé		P					3	GC		
*Coryphopterusdicrus* Bohlke & Robins, 1960	Colon Goby			P		Yes			3	WA		
*Coryphopteruseidolon* Bohlke & Robins, 1960	Pallid Goby			P		Yes			3	GC		
*Coryphopterusglaucofraenum* Gill, 1863	Bridled Goby					Yes				WA		
*Coryphopterushyalinus* Bohlke & Robins, 1962	Glass Goby			P		Yes			4	GC		
*Coryphopteruskuna* Victor, 2007	Kuna Goby	Estapé		P					4	GC		
*Coryphopteruslipernes* Bohlke & Robins, 1962	Peppermint Goby			P		Yes			4	GC		
*Coryphopteruspersonatus* (Jordan & Thompson, 1905)	Masked Goby		V	P		Yes			4	GC		
*Coryphopterusthrix* Bohlke & Robins, 1960	Bartail Goby			P		Yes			4	WA		
*Coryphopterustortugae* (Jordan, 1904)	Sand Goby			P		Yes			4	GC		
*Coryphopterusvenezuelae* Cervigon, 1966	Sand-Canyon Goby	Estapé		P					4	GC		
*Ctenogobiussaepepallens* (Gilbert & Randall, 1968)	Dash Goby	Estapé		P					4	GC		
*Elacatinuschancei* (Beebe & Hollister, 1933)	Shortstripe Goby		C	P		Yes	Yes		4	GC	L	
*Elacatinusevelynae* (Bohlke & Robins, 1968)	Sharknose Goby			P		Yes	Yes		4	GC		
Genus 1 species 5		DROP	CP						13	GC?	?	Yes
Genus 1 species 6		DROP	CP						13	GC?	?	Yes
Genus 2 species 1		DROP	CP						13	GC?	?	Yes
*Ginsburgellusnovemlineatus* (Fowler, 1950)	Ninelined Goby	Estapé		P					4	GC		
*Gnatholepisthompsoni* Jordan, 1904	Goldspot Goby		V	P		Yes	Yes		4	TA		
*Lythrypnuselasson* Bohlke & Robins, 1960	Dwarf Goby	DROP/ Estapé	C	P					4	GC		
*Microgobiuscarri* Fowler, 1945	Seminole Goby	Estapé		P					4	WA		
*Neslongus* (Nichols, 1914)	Orangespotted Goby			P		Yes			4	GC		
*Palatogobiusgrandoculus* Greenfield, 2002	Bigeye Goby	DROP	CP				DROP		4	GC		Yes
*Palatogobiusincendius* Tornabene, Robertson & Baldwin, 2017	Ember Goby	DROP	C				DROP			GC		Yes
*Pinnichthysaimoriensis* Van Tassell & Tornabene, 2016	Thiony’s Goby	DROP	CP						4	GC		Yes
*Priolepishipoliti* (Metzelaar, 1922)	Rusty Goby			P		Yes			4	WA		
*Ptereleotrishelenae* (Randall, 1968)	Hovering Dartfish		V	P		Yes			4	GC		
*Risorruber* (Rosen, 1911)	Tusked Goby		C	P		Yes	Yes		4	WA		
*Tigrigobiusdilepis* (Robins & Bohlke, 1964)	Orangesided Goby			P		Yes			4	GC		
*Tigrigobiusmultifasciatus* (Steindachner, 1876)	Greenbanded Goby	Estapé		P					4	GC	L	
*Varicuscephalocellatus* Gilmore, Van Tassell & Baldwin, 2016	Ocellated Split-Fin Goby	DROP	CP				DROP		4	GC	L	Yes
*Varicusveliguttatus* Van Tassell, Baldwin & Gilmore, 2016	Spotted-Sail Goby	DROP	CP				DROP		4	GC		Yes
GRAMMATIDAE												
*Grammalinki* Starck & Colin, 1978	Yellowcheek Basslet	DROP	CP				DROP		5	GC		
*Grammaloreto* Poey, 1868	Fairy Basslet			P	Yes	Yes			5	GC		
*Lipogrammaevides* Robins & Colin, 1979	Banded Basslet	DROP	CP				DROP		5	GC		Yes
*Lipogrammaklayi* Randall, 1963	Bicolor Basslet	DROP	CP						5	GC		Yes
*Lipogrammalevinsoni* Baldwin, Nonaka & Robertson, 2016	Hourglass Basslet	DROP	CP						5	GC		Yes
*Lipogrammaregia* Robins & Colin, 1979	Royal Basslet	DROP	CP				DROP		5	GC		Yes
*Lipogrammatrilineata* Randall, 1963	Threeline Basslet	DROP	CP				DROP		5	GC		Yes
GRAMMICOLEPIDIDAE												
*Grammicolepisbrachiusculus* Poey, 1873	Thorny Tinselfish					Yes				PAN		Yes
HAEMULIDAE												
*Anisotremussurinamensis* (Bloch, 1791)	Black Margate			P	Yes	Yes			5	WA		
*Brachygenyschrysargyreum* (Gunther, 1859)	Smallmouth Grunt			P		Yes	Yes	Yes	5	GC		
*Haemulonalbum* Cuvier, 1830	Margate			P	Yes	Yes			5	WA		
*Haemulonaurolineatum* Cuvier, 1830	Tomtate			P	Yes	Yes	Yes		5	WA		
*Haemuloncarbonarium* Poey, 1860	Caesar Grunt			P	Yes	Yes	Yes	Yes	5	GC		
*Haemulonflavolineatum* (Desmarest, 1823)	French Grunt			P	Yes	Yes	Yes	Yes	5	GC		
*Haemulonmacrostomum* Gunther, 1859	Spanish Grunt					Yes				GC		
*Haemulonmelanurum* (Linnaeus, 1758)	Cottonwick			P		Yes			5	WA		
*Haemulonparra* (Desmarest, 1823)	Sailors Choice					Yes				WA		
*Haemulonplumierii* (Lacepede, 1801)	White Grunt			P		Yes			5	WA		
*Haemulonsciurus* (Shaw, 1803)	Bluestriped Grunt			(P)		Yes	Yes		5	GC		
*Haemulonstriatum* (Linnaeus, 1758)	Striped Grunt		V	V		Yes				WA		
*Haemulonvittatum* (Poey, 1860)	Boga			P		Yes			5	GC		
HALOSAURIDAE												
*Halosaurusovenii* Johnson, 1864	Stripejaw Halosaur					Yes				TA,IWP		Yes
HEMIRAMPHIDAE												
*Hemiramphusbrasiliensis* (Linnaeus, 1758)	Ballyhoo			P		Yes			5	WA		
HOLOCENTRIDAE												
*Cornigerspinosus* Agassiz, 1831	Spinycheek Soldierfish	DROP	V							TA		Yes
*Holocentrusadscensionis* (Osbeck, 1765)	Squirrelfish		V	P	Yes	Yes	Yes		5	TA		
*Holocentrusrufus* (Walbaum, 1792)	Longspine Squirrelfish		V	P	Yes	Yes	Yes		5	GC		
*Myripristisjacobus* Cuvier, 1829	Blackbar Soldierfish		V	P		Yes	Yes		5	TA		
*Neoniphoncoruscum* (Poey, 1860)	Reef Squirrelfish			P		Yes			5	GC		
*Neoniphonmarianus* (Cuvier, 1829)	Longjaw Squirrelfish		C	P		Yes	Yes		5	GC		
*Neoniphonvexillarium* (Poey, 1860)	Dusky Squirrelfish			P		Yes			5	GC		
*Ostichthystrachypoma* (Gunther, 1859)	Bigeye Soldierfish	DROP	CP				DROP		6	WA		Yes
*Plectrypopsretrospinis* (Guichenot, 1853)	Cardinal Soldierfish	Estapé		P					6	WA		
ISTIOPHORIDAE												
*Istiophorusplatypterus* (Shaw, 1792)	Sailfish					Yes				TA		
*Makairanigricans* Lacepede, 1802	Blue Marlin					Yes				PAN		
KYPHOSIDAE												
*Kyphosusbigibbus* Lacepede, 1801	Gray Seachub	Estapé		P					6	TA/IWP		
*Kyphosuscinerascens* (Forsskal, 1775)	Topsail Seachub			P		Yes			6	PAN		
*Kyphosussectatrix* (Linnaeus, 1766)	Bermuda Chub			P		Yes			6	PAN		
*Kyphosusvaigiensis* (Quoy & Gaimard, 1825)	Yellow Chub			V		Yes				PAN		
LABRIDAE												
Labrinae												
*Bodianusrufus* (Linnaeus, 1758)	Spanish Hogfish		V	P	Yes	Yes		Yes	6	WA		
*Clepticusparrae* (Bloch & Schneider, 1801)	Creole Wrasse		V	P	Yes	Yes	Yes		6	GC		
*Decodonpuellaris* (Poey, 1860)	Red Hogfish	DROP	CP				DROP		6	WA		Yes
*Decodon* species 2		DROP	CP						13	GC		Yes
*Halichoeresbathyphilus* (Beebe & Tee-Van,1932)	Greenband Wrasse	DROP	V							GC		Yes
*Halichoeresbivittatus* (Bloch, 1791)	Slippery Dick			P	Yes	Yes			6	WA		
*Halichoerescyanocephalus* (Bloch, 1791)	Yellowcheek Wrasse			P	Yes	Yes			6	GC		
*Halichoeresgarnoti* (Valenciennes, 1839)	Yellowhead Wrasse		V	P	Yes	Yes	Yes		6	GC		
*Halichoeresmaculipinna* (Müller & Troschel, 1848)	Clown Wrasse			P	Yes	Yes			6	GC		
*Halichoerespictus* (Poey, 1860)	Rainbow Wrasse			P		Yes			6	GC		
*Halichoerespoeyi* (Steindachner, 1867)	Blackear Wrasse			P	Yes	Yes			6	WA		
*Halichoeresradiatus* (Linnaeus, 1758)	Puddingwife			P	Yes	Yes			6	WA		
*Thalassomabifasciatum* (Bloch, 1791)	Bluehead		V	P	Yes	Yes	Yes		6	GC		
*Xyrichtysmartinicensis* Valenciennes, 1840	Rosy Razorfish			P	Yes	Yes			6	GC		
*Xyrichtysnovacula* (Linnaeus, 1758)	Pearly Razorfish			P		Yes			6	WA		
*Xyrichtyssplendens* Castelnau, 1855	Green Razorfish			P	Yes	Yes	Yes		6	GC		
Scarinae												
*Cryptotomusroseus* Cope, 1871	Bluelip Parrotfish			P		Yes			6	WA		
*Scaruscoeruleus* (Bloch, 1786)	Blue Parrotfish				Yes	Yes				GC		
*Scarusguacamaia* Cuvier, 1829	Rainbow Parrotfish					Yes				GC		
*Scarusiseri* (Bloch, 1789)	Striped Parrotfish			P	Yes	Yes		Yes	6	GC		
*Scarustaeniopterus* Desmarest, 1831	Princess Parrotfish		V	P	Yes	Yes	Yes	Yes	6	GC		
*Scarusvetula* Bloch & Schneider, 1801	Queen Parrotfish			P	Yes	Yes	Yes	Yes	6	GC		
*Sparisomaatomarium* (Poey, 1861)	Greenblotch Parrotfish			P		Yes			6	GC		
*Sparisomaaurofrenatum* (Valenciennes, 1840)	Redband Parrotfish		V	P	Yes	Yes	Yes	Yes	7	GC		
*Sparisomachrysopterum* (Bloch & Schneider, 1801)	Redtail Parrotfish			P	Yes	Yes		Yes	7	GC		
*Sparisomaradians* (Valenciennes, 1840)	Bucktooth Parrotfish			P		Yes			7	WA		
*Sparisomarubripinne* (Valenciennes, 1840)	Yellowtail Parrotfish			P		Yes		Yes	7	GC		
*Sparisomaviride* (Bonnaterre, 1788)	Stoplight Parrotfish		V	P	Yes	Yes		Yes	7	GC		
LABRISOMIDAE												
*Brockiusnigricinctus* Howell Rivero, 1936	Spotcheek Blenny	Estapé		P					7	GC		
*Gobioclinusbucciferus* Poey, 1868	Puffcheek Blenny	Estapé		P					7	GC		
*Gobioclinusgobio* (Valenciennes, 1836)	Palehead Blenny	Estapé		P					7	WA		
*Gobioclinusguppyi* (Norman, 1922)	Mimic Blenny	Estapé		P					7	WA		
*Labrisomusnuchipinnis* (Quoy & Gaimard, 1824)	Hairy Blenny			P	Yes	Yes			7	TA		
*Malacoctenusaurolineatus* Smith, 1957	Goldline Blenny			P		Yes			7	GC		
*Malacoctenusboehlkei* Springer, 1959	Diamond Blenny					Yes				GC		
*Malacoctenuserdmani* Smith, 1957	Imitator Blenny	Estapé		P					7	GC		
*Malacoctenusmacropus* (Poey, 1868)	Rosy Blenny	Estapé		P					7	GC		
*Malacoctenustriangulatus* Springer, 1959	Saddled Blenny			P		Yes			7	GC		
LOBOTIDAE												
*Lobotessurinamensis* (Bloch, 1790)	Atlantic Tripletail					Yes				TA/IWP		
LOPHIIDAE												
*Lophiodesmonodi* Le Danois, 1971	Club-bait Goosefish					Yes				GC		Yes
LUTJANIDAE												
*Apsilusdentatus* Guichenot, 1853	Black Snapper					Yes				GC		Yes
*Etelisoculatus* (Valenciennes, 1828)	Queen Snapper					Yes				WA		Yes
*Lutjanusanalis* (Cuvier, 1828)	Mutton Snapper			P	Yes	Yes			7	WA		
*Lutjanusapodus* (Walbaum, 1792)	Schoolmaster		V	P	Yes	Yes	Yes	Yes	7	GC		
*Lutjanusbuccanella* (Cuvier, 1828)	Blackfin Snapper		V	P		Yes			7	WA		
*Lutjanuscyanopterus* (Cuvier, 1828)	Cubera Snapper			P		Yes			7	WA		
*Lutjanusgriseus* (Linnaeus, 1758)	Gray Snapper			(P)	Yes	Yes			7	TA		
*Lutjanusjocu* (Bloch & Schneider, 1801)	Dog Snapper			P	Yes	Yes			7	TA		
*Lutjanusmahogoni* (Cuvier, 1828)	Mahogany Snapper		V	P	Yes	Yes	Yes	Yes	7	GC		
*Lutjanuspurpureus* (Poey, 1866)	Caribbean Red Snapper					Yes				TA		
*Lutjanussynagris* (Linnaeus, 1758)	Lane Snapper			P	Yes	Yes	Yes		7	TA		
*Lutjanusvivanus* (Cuvier, 1828)	Silk Snapper					Yes				TA		Yes
*Ocyuruschrysurus* (Bloch, 1791)	Yellowtail Snapper			P	Yes	Yes		Yes	7	TA		
*Pristipomoides* sp.^1^			V							WA?	?	Yes
MACROURIDAE												
*Gadomusarcuatus* (Goode & Bean, 1886)	Doublethread Grenadier					Yes				TA		Yes
*Gadomusdispar* (Vaillant, 1888)	Onelong Grenadier					Yes				TA		Yes
*Hymenocephalusaterrimus* Gilbert, 1905	Nobeard Grenadier					Yes				WA/PAC		Yes
*Hymenocephalusbillsam* Marshall & Iwamoto, 1973	Bigeye Grenadier					Yes				WA		Yes
*Malacocephaluslaevis* (Lowe, 1843)	Velvet Grenadier					Yes				PAN		Yes
*Nezumiaaequalis* (Günther, 1878)	Atlantic Blacktip Grenadier					Yes				TA		Yes
*Ventrifossamacropogon* Marshall, 1973	Longbeard Grenadier					Yes				WA/WPAC		Yes
MALACANTHIDAE												
*Malacanthusplumieri* (Bloch, 1786)	Sand Tilefish		V	P	Yes	Yes	Yes		7	WA		
MEGALOPIDAE												
*Megalopsatlanticus* Valenciennes, 1847	Tarpon			P		Yes			8	TA		
MERLUCCIIDAE												
*Steindachneriaargentea* Goode & Bean, 1896	Luminous Hake					Yes				GC		Yes
MONACANTHIDAE												
*Aluterusscriptus* (Osbeck, 1765)	Scrawled Filefish			P	Yes	Yes			8	PAN		
*Cantherhinesmacrocerus* (Hollard, 1853)	Whitespotted Filefish			P	Yes	Yes	Yes	Yes	8	WA		
*Cantherhinespullus* (Ranzani, 1842)	Orangespotted Filefish			P	Yes	Yes		Yes	8	TA		
*Monacanthusciliatus* (Mitchill, 1818)	Fringed Filefish			P	Yes	Yes			8	TA		
*Monacanthustuckeri* Bean, 1906	Slender Filefish			P	Yes	Yes			8	GC		
*Stephanolepissetifer* (Bennett, 1831)	Pygmy Filefish			P	Yes	Yes			8	WA		
MUGILIDAE												
*Mugilcurema* Valenciennes, 1836	White Mullet					Yes				TA		
MULLIDAE												
*Mulloidichthysmartinicus* (Cuvier, 1829)	Yellow Goatfish		V	P	Yes	Yes	Yes		8	TA		
*Pseudupeneusmaculatus* (Bloch, 1793)	Spotted Goatfish		V	P	Yes	Yes	Yes		8	WA		
MURAENIDAE												
*Echidnacatenata* (Bloch, 1795)	Chain Moray			P		Yes			8	WA		
*Enchelycorecarychroa* Bohlke & Bohlke, 1976	Chestnut Moray	Estapé		(P)					8	TA		
*Enchelycorenigricans* (Bonnaterre, 1788)	Viper Moray	Estapé		(P)					8	TA		
*Gymnothoraxfunebris* Ranzani, 1839	Green Moray			P	Yes	Yes			8	TA		
*Gymnothoraxmiliaris* (Kaup, 1856)	Goldentail Moray			P		Yes	Yes		8	TA		
*Gymnothoraxmoringa* (Cuvier, 1829)	Spotted Moray			P	Yes	Yes	Yes		8	TA		
*Gymnothoraxvicinus* (Castelnau, 1855)	Purplemouth Moray			(P)		Yes			8	TA		
NARCINIDAE												
*Narcinebancroftii* (Griffith & Smith, 1834)	Lesser Electric Ray					Yes				GC		
OGCOCEPHALIDAE												
*Dibranchusatlanticus* Peters, 1876	Atlantic Batfish					Yes				TA		Yes
*Ogcocephaluscorniger* Bradbury, 1980	Longnose Batfish	DROP	CP						8	GC		
*Zalieutesmcgintyi* (Fowler, 1952)	Tricorn Batfish	DROP	CP						8	GC		Yes
OPHICHTHIDAE												
*Myrichthysbreviceps* (Richardson, 1848)	Sharptail Eel					Yes				WA		
*Myrichthysocellatus* (Lesueur, 1825)	Goldspotted Eel	Estapé		P					8	WA		
*Ophichthusophis* (Linnaeus, 1758)	Spotted Snake Eel					Yes				WA		
OPHIDIIDAE												
*Brotulabarbata* (Bloch & Schneider, 1801)	Atlantic Bearded Brotula	DROP	CP				DROP		8	TA		
*Neobythiteselongatus* Nielsen & Retzler, 1994	Elongate Cusk-eel					Yes				GC		Yes
*Parophidionschmidti* (Woods & Kanazawa, 1951)	Dusky Cusk-eel	Estapé		P					8	GC		
OPISTOGNATHIDAE												
*Opistognathusaurifrons* (Jordan & Thompson, 1905)	Yellowhead Jawfish			P	Yes	Yes	Yes		8	WA		
*Opistognathusmacrognathus* Poey, 1860	Banded Jawfish					Yes				GC		
*Opistognathusmaxillosus* Poey, 1860	Mottled Jawfish	Estapé		P					8	GC		
OSTRACIIDAE												
*Acanthostracionpolygonius* Poey, 1876	Honeycomb Cowfish		V	P	Yes	Yes	Yes		8	WA		
*Acanthostracionquadricornis* (Linnaeus, 1758)	Scrawled Cowfish		V	P		Yes			9	TA		
*Lactophrysbicaudalis* (Linnaeus, 1758)	Spotted Trunkfish			P		Yes	Yes		9	TA		
*Lactophrystrigonus* (Linnaeus, 1758)	Trunkfish			P	Yes	Yes			9	TA		
*Lactophrystriqueter* (Linnaeus, 1758)	Smooth Trunkfish			P	Yes	Yes	Yes		9	WA		
PARALICHTHYIDAE												
*Citharichthyscornutus* (Gunther, 1880)	Horned Whiff					Yes				WA		Yes
*Gastropsettafrontalis* Bean, 1895	Shrimp Flounder	DROP	CP				DROP		9	GC		
*Syaciummicrurum* Ranzani, 1842	Channel Flounder			P		Yes			9	WA		
PARAZENIDAE												
*Cyttopsisrosea* (Lowe, 1843)	Red Dory					Yes				TA/IWP		Yes
PEMPHERIDAE												
*Pempherisschomburgkii* Müller & Troschel, 1848	Glassy Sweeper			P		Yes			9	WA		
PENTANCHIDAE												
*Apristuruscanutus* Springer & Heemstra, 1979	Hoary Cat Shark					Yes				GC		Yes
*Galeusantillensis* Springer, 1979	Antilles Sawtail Catshark					Yes				GC	L	Yes
PERCOPHIDAE												
*Bembropsocellatus* Thompson & Suttkus, 1998	Ocellate Duckbill					Yes				GC		Yes
*Bembropsquadrisella* Thompson & Suttkus, 1998	Saddleback Duckbill					Yes				GC		Yes
*Chrionemasquamentum* (Ginsburg, 1955)	Scalychin Flathead	DROP	CP				DROP		9	GC		Yes
PERISTEDIIDAE												
*Peristediontruncatum* (Gunther, 1880)	Black Armored Searobin					Yes				WA		Yes
POLYMIXIIDAE												
*Polymixialowei* Gunther, 1859	Beardfish					Yes				WA		Yes
POMACANTHIDAE												
*Centropygeargi* Woods & Kanazawa, 1951	Cherubfish		V	P		Yes			9	GC		
*Holacanthusciliaris* (Linnaeus, 1758)	Queen Angelfish		V	P	Yes	Yes		Yes	9	WA		
*Holacanthustricolor* (Bloch, 1795)	Rock Beauty		V	P	Yes	Yes	Yes	Yes	9	WA		
*Pomacanthusarcuatus* (Linnaeus, 1758)	Gray Angelfish				Yes	Yes		Yes		WA		
*Pomacanthusparu* (Bloch, 1787)	French Angelfish		V	P	Yes	Yes	Yes	Yes	9	WA		
POMACENTRIDAE												
*Abudefdufsaxatilis* (Linnaeus, 1758)	Sergeant Major			P	Yes	Yes			9	TA		
*Abudefduftaurus* (Müller & Troschel, 1848)	Night Sergeant			P		Yes			9	TA		
Chromiscf.enchrysura ^2^		DROP	CP				DROP		13	WA		Yes
*Chromiscyanea* (Poey, 1860)	Blue Chromis		V	P	Yes	Yes	Yes		9	GC		
*Chromisinsolata* (Cuvier, 1830)	Sunshinefish		V	P		Yes			9	GC		
*Chromismultilineata* (Guichenot, 1853)	Brown Chromis		V	P	Yes	Yes	Yes		9	TA		
*Chromisscotti* Emery, 1968	Purple Reeffish	DROP	V							WA		
*Microspathodonchrysurus* (Cuvier, 1830)	Yellowtail Damselfish			P	Yes	Yes	Yes	Yes	9	WA		
*Stegastesadustus* (Troschel, 1865)	Dusky Damselfish			P		Yes			9	GC		
*Stegastesdiencaeus* (Jordan & Rutter, 1897)	Longfin Damselfish			P		Yes			9	GC		
*Stegastesleucostictus* (Müller & Troschel, 1848)	Beaugregory				Yes	Yes				GC		
*Stegastespartitus* (Poey, 1868)	Bicolor Damselfish		V	P	Yes	Yes	Yes		9	GC		
*Stegastesplanifrons* (Cuvier, 1830)	Threespot Damselfish			P		Yes			9	GC		
*Stegastesxanthurus* (Poey, 1860)	Cocoa Damselfish			P		Yes			9	GC		
PRIACANTHIDAE												
*Heteropriacanthuscruentatus* (Lacepède, 1801)	Glasseye Snapper			P		Yes	Yes		9	TA		
*Priacanthusarenatus* Cuvier, 1829	Bigeye		V			Yes				TA		
*Pristigenysalta* (Gill, 1862)	Short Bigeye	DROP	V							WA		
RHINCODONTIDAE												
*Rhincodontypus* Smith, 1828	Whale Shark					Yes				PAN		
SCIAENIDAE												
*Equetuslanceolatus* (Linnaeus, 1758)	Jackknife-fish		V	P	Yes	Yes			10	WA		
*Equetuspunctatus* (Bloch & Schneider, 1801)	Spotted Drum			P		Yes	Yes		10	WA		
*Parequesacuminatus* (Bloch & Schneider, 1801)	High-hat			P		Yes			10	WA		
*Umbrinacoroides* Cuvier, 1830	Sand Drum					Yes				WA		
SCOMBRIDAE												
*Acanthocybiumsolandri* (Cuvier, 1832)	Wahoo					Yes				PAN		
*Euthynnusalletteratus* (Rafinesque, 1810)	Little Tunny			P		Yes			10	TA		
*Katsuwonuspelamis* (Linnaeus, 1758)	Skipjack Tuna					Yes				PAN		
*Scomberomoruscavalla* (Cuvier, 1829)	King Mackerel			P		Yes			10	WA		
*Scomberomorusregalis* (Bloch, 1793)	Cero		V	P	Yes	Yes			10	WA		
*Thunnusatlanticus* (Lesson, 1831)	Blackfin Tuna					Yes				WA		
SCORPAENIDAE												
*Pontinuscastor* Poey, 1860	Longsnout Scorpionfish	DROP	CP				DROP		10	GC		Yes
*Pontinusnematophthalmus* (Gunther, 1860)	Spinythroat Scorpionfish	DROP	CP						10	WA		Yes
*Pteroisvolitans* (Linnaeus, 1758)	Red Lionfish		V	P		Yes	Yes		10	NA	NA	NA
*Scorpaenaplumieri* Bloch, 1789	Spotted Scorpionfish			P		Yes	Yes		10	TA		
*Scorpaenodescaribbaeus* Meek & Hildebrand, 1928	Reef Scorpionfish	Estapé		P					10	WA		
SERRANIDAE												
*Alphestesafer* (Bloch, 1793)	Mutton Hamlet			(P)		Yes			10	TA		
*Baldwinellavivanus* (Jordan & Swain, 1885)^3^	Red Barbier	DROP	V							WA		Yes
*Bathyanthias* species A		DROP	CP						13	GC	L	Yes
*Bullisichthyscaribbaeus* Rivas, 1971	Pugnose Bass	DROP	CP				DROP		10	GC		Yes
*Cephalopholiscruentata* (Lacepede, 1802)	Graysby		V	P	Yes	Yes	Yes	Yes	10	GC		
*Cephalopholisfulva* (Linnaeus, 1758)	Coney		V	P	Yes	Yes	Yes	Yes	10	WA		
*Diplectrumbivittatum* (Valenciennes, 1828)	Dwarf Sand Perch	Estapé		P					10	WA		
*Epinephelusadscensionis* (Osbeck, 1765)	Rock Hind		V	(P)		Yes			10	TA		
*Epinephelusguttatus* (Linnaeus, 1758)	Red Hind		V	P	Yes	Yes	Yes	Yes	10	WA		
*Epinephelusstriatus* (Bloch, 1792)	Nassau Grouper					Yes				GC		
*Gonioplectrushispanus* (Cuvier, 1828)	Spanish Flag	DROP	V							WA		Yes
*Hypoplectruschlorurus* (Cuvier, 1828)	Yellowtail Hamlet			P	Yes	Yes			10	GC		
*Hypoplectrusguttavarius* (Poey, 1852)	Shy Hamlet			P		Yes			10	GC		
*Hypoplectrusindigo* (Poey, 1851)	Indigo Hamlet	DROP	V							GC		
*Hypoplectrusnigricans* (Poey, 1852)	Black Hamlet					Yes				GC		
*Hypoplectruspuella* (Cuvier, 1828)	Barred Hamlet		V	P	Yes	Yes	Yes		10	GC		
*Hypoplectrus* species 1	Bluelip Hamlet	Estapé		P					13	GC		
*Hypoplectrusunicolor* (Walbaum, 1792)	Butter Hamlet					Yes				GC		
*Liopropomacarmabi* (Randall, 1963)	Candy Basslet	DROP	CP				DROP		10	WA		
*Liopropomamowbrayi* Woods & Kanazawa, 1951	Cave Basslet	DROP	CP				DROP		10	GC		
*Liopropomaolneyi* Baldwin & Johnson, 2014	Yellow-Spotted Basslet	DROP	CP				DROP		10	GC	L	Yes
*Liopropomarubre* Poey, 1861	Peppermint Basslet			P		Yes			11	GC		
*Mycteropercainterstitialis* (Poey, 1860)	Yellowmouth Grouper					Yes				WA		
*Mycteropercatigris* (Valenciennes, 1833)	Tiger Grouper					Yes				WA		
*Mycteropercavenenosa* (Linnaeus, 1758)	Yellowfin Grouper			P	Yes	Yes			11	WA		
*Paranthiasfurcifer* (Valenciennes, 1828)	Atlantic Creolefish		V	P	Yes	Yes			11	TA		
*Plectranthias* species A		DROP	CP						13	GC	L	Yes
*Pronotogrammusmartinicensis* (Guichenot, 1868)	Roughtongue Bass	DROP	CP				DROP		11	WA		Yes
*Rypticusbistrispinus* (Mitchill, 1818)	Freckled Soapfish					Yes				WA		
*Rypticussaponaceus* (Bloch & Schneider, 1801)	Greater Soapfish		V	P	Yes	Yes	Yes		11	TA		
*Serranusannularis* (Gunther, 1880)	Orangeback Bass	DROP	V							WA		
*Serranusbaldwini* (Evermann & Marsh, 1899)	Lantern Bass			P	Yes	Yes	Yes		11	WA		
*Serranusflaviventris* (Cuvier, 1829)	Twinspot Bass					Yes				WA		
*Serranusfuscula* (Poey, 1861)	Twospot Sea Bass	DROP	CP				DROP		11	WA		Yes
*Serranusluciopercanus* Poey, 1852	Crosshatch Bass	DROP	V							GC		Yes
*Serranusnotospilus* Longley, 1935	Saddle Bass	DROP	V							GC		
*Serranusphoebe* Poey, 1851	Tattler		V			Yes				WA		Yes
*Serranustabacarius* (Cuvier, 1829)	Tobaccofish		V	P	Yes	Yes	Yes		11	WA		
*Serranustigrinus* (Bloch, 1790)	Harlequin Bass		V	P	Yes	Yes	Yes		11	GC		
*Serranustortugarum* Longley, 1935	Chalk Bass		V	P		Yes	Yes		11	GC		
SETARCHIDAE												
*Setarchesguentheri* Johnson, 1862	Deepwater Scorpionfish					Yes				TA/IWP		Yes
SPARIDAE												
*Calamusbajonado* (Bloch & Schneider, 1801)	Jolthead Porgy				Yes	Yes				WA		
*Calamuscalamus* (Valenciennes, 1830)	Saucereye Porgy			P	Yes	Yes			11	WA		
*Calamuspennatula* Guichenot, 1868	Pluma Porgy			P		Yes			11	WA		
SPHYRAENIDAE												
*Sphyraenabarracuda* (Edwards, 1771)	Great Barracuda		V	P	Yes	Yes	Yes	Yes	11	PAN		
*Sphyraenaborealis* DeKay, 1842	Sennet			P		Yes			11	WA		
SPHYRNIDAE												
*Sphyrnamokarran* (Rüppell, 1837)	Great Hammerhead					Yes				PAN		
SQUALIDAE												
*Squalusclarkae* Pfleger, Grubbs, Cotton & Daly-Engel, 2018	Gulf Dogfish					Yes				GC		Yes
SYMPHYSANODONTIDAE												
*Symphysanodonberryi* Anderson, 1970	Slope Bass	DROP	CP				DROP		11	TA		Yes
*Symphysanodonoctoactinus* Anderson, 1970	Insular Bunquelovely	DROP	CP				DROP		11	GC		Yes
SYNGNATHIDAE												
*Amphelikturusdendriticus* (Barbour, 1905)	Seahorse Pipefish	Estapé		P					11	WA		
*Bryxdunckeri* (Metzelaar, 1919)	Pugnose Pipefish					Yes				WA		
*Cosmocampusalbirostris* (Kaup, 1856)	Whitenose Pipefish					Yes				WA		
*Halicampuscrinitus* (Jenyns, 1842)	Banded Pipefish	Estapé		V						WA		
*Hippocampuserectus* Perry, 1810	Lined Seahorse			P			Yes		11	WA		
*Hippocampusreidi* Ginsburg, 1933	Longsnout Seahorse			P		Yes			11	GC		
SYNODONTIDAE												
*Synodusfoetens* (Linnaeus, 1766)	Inshore Lizardfish					Yes				NWA		
*Synodusintermedius* (Agassiz, 1829)	Sand Diver			P	Yes	Yes	Yes		11	WA		
*Synodussynodus* (Linnaeus, 1758)	Red Lizardfish			P		Yes			11	TA		
*Trachinocephalusmyops* (Forster, 1801)	Snakefish			P		Yes			11	TA		
TETRAODONTIDAE												
*Canthigasterjamestyleri* Moura & Castro, 2002	Goldface Toby	DROP	CP				DROP		11	GC		
*Canthigasterrostrata* (Bloch, 1786)	Sharpnose Puffer		V	P	Yes	Yes	Yes		11	GC		
*Sphoeroidesdorsalis* Longley, 1934	Marbled Puffer	DROP/ Estapé	CP	P			DROP		12	GC		
*Sphoeroidesnephelus* (Goode & Bean, 1882)	Southern Puffer					Yes				GC		
*Sphoeroidesspengleri* (Bloch, 1785)	Bandtail Puffer			P	Yes	Yes			12	WA		
TRACHICHTHYIDAE												
*Hoplostethusoccidentalis* Woods, 1973	Western Roughy					Yes				WA		Yes
TRIACANTHODIDAE												
*Hollardiahollardi* Poey, 1861	Reticulate Spikefish					Yes				GC		Yes
TRIGLIDAE												
*Bellatoregretta* (Goode & Bean, 1896)	Streamer Searobin	DROP	CP						12	GC		Yes
TRIPTERYGIIDAE												
*Enneanectesaltivelis* Rosenblatt, 1960	Lofty Triplefin	Estapé		P					12	GC		
*Enneanectesboehlkei* Rosenblatt, 1960	Roughhead Triplefin	Estapé		P					12	GC		
*Enneanectesjordani* (Evermann & Marsh, 1899)	Mimic Triplefin			p		Yes			12	GC		
*Enneanectesmatador* Victor, 2013	Matador Triplefin	Estapé		p					12	GC		

Notes: 1. *Pristipomoides*. This is *P.aquilonaris* and/or *P.macrophthalmus*. Statia is within the geographical range of both species. 2. Chromiscf.enchrysura is an undescribed species recorded as *C.enchrysura* in the GBIF database, where it is a DROP entry. 3. The *Baldwinella* “*vivanus*” population from the Caribbean likely is a separate species from *B.vivanus*, which was described from specimens collected on the north coast of Cuba. Photograph credits: B Brown: *A.nikkiae*, *B.barbata*, *C.jamestyleri*, *D.puellaris*, *D.lombardii*, *Foetorepus* sp, *G.linkii*, *L.mowbrayi*, *L.klayi*, *L.regia*, *P.grandoculus*, *S.fuscula*, *V.cephalocellatus*, *Z.mcgyntii*; M and R Bentley: *A.narinari*, *E.carychroa*, *E.adscensionis*, *G.cirratum*, *L.griseus*, *H.sciurus*; M Harterink: *A.balearicum*, *E.carib*, *E.vitta*, *E.nigricans*, *G.vicinus*, *H.exstochilus*; M Pistor (STENAPA): *A.afer*, *H.histrio*; all other photographs are by the two sets of coauthors during their respective expeditions in 2017 and 2020.

### Photographic plates

The 13 photographic plates (Suppl. materials 4–16: Plates S1–S13) include images of 280 species, 69% of those on the Statia20 list. In addition, Davies and Piontek (2017) provided images of *Chimaeracubana*, which are not included in the supplemental plates. Of the plate images, 40 species come from DROP collections, 226 were taken by CJE and AME and 14 were provided to them by local divers and fishers at Statia (Table 1). Images are available from other sources for all remaining species listed in Table 1 (except the seven species of macrourids), on their individual species pages at https://biogeodb.stri.si.edu/caribbean/en/pages.

## Structure of the Statia20 reef-associated bony fish fauna

### Global geographical ranges

**Figure 3.**
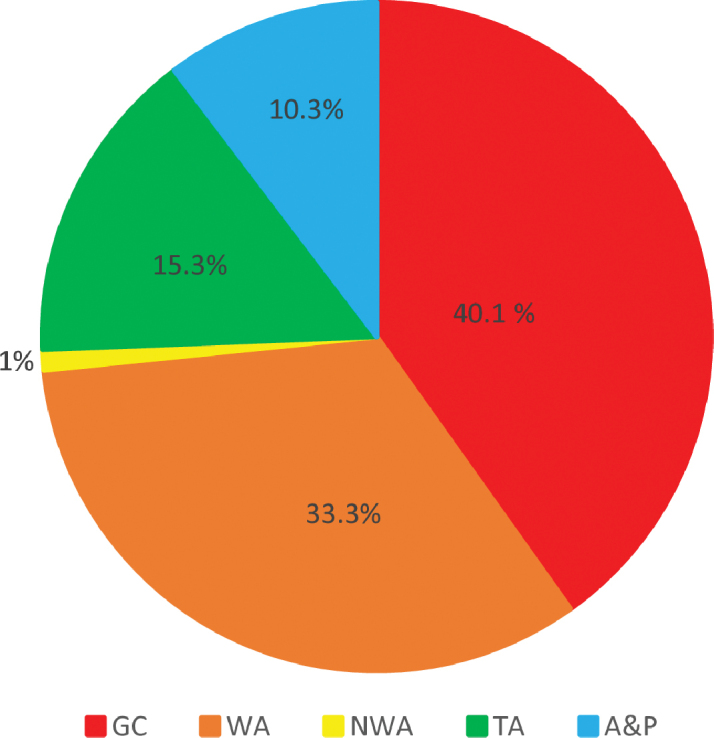
Percentages of the Sint Eustatius marine fish fauna represented by groups of species with different global geographical ranges. GC = Greater Caribbean endemics; NWA = GC plus temperate eastern USA; WA = GC plus Brazil; TA = WA plus central or East Atlantic; and A&P = species found in both the Atlantic and various parts of the Indo-Pacific.

Greater Caribbean endemics represent the largest group of species in the Statia fauna, and, together with more widely ranging western Atlantic endemics, constitute almost three quarters of the species. Trans-Atlantic species and species found outside as well as inside the Atlantic represented only a quarter of the fauna (Table 1, Figure 3). The relative abundances of species with different types of large-scale geographic ranges are very similar to those of species in the well documented fauna of nearby St. Croix (Smith-Vaniz and Jelks 2014). Species found in Brazil constituted one third of the Statia fauna, while those extending northwards from the Greater Caribbean represented only 1%, a reflection of the greater effects of temperature limitation on northward extension of ranges as compared to effects of the Amazon-Orinoco outflow on limitation of range extension much further south of the Greater Caribbean.

### Extent of geographical ranges within the Greater Caribbean

The vast majority of species are widely distributed within the Greater Caribbean, with only nine (2.25%) of them having ranges limited to a restricted part of the Caribbean. Among those nine, five are deep-living species, and five belong to Core CRF families (Table 1). The four shallow species with restricted ranges are all Core CRFs. None of the species were micro-endemics, restricted to Statia or that island plus immediately surrounding islands, and no micro-endemics are known to exist in that general area.

### Ecology – Depth

The number of deep species increased from 44 on the Statia17 list to 86 in the Statia20 fauna (Table 1), representing an increase from 14.5% in the former to 21.2% in the latter. Among the reef-associated bony fishes (Table 2) the number of deep species increased from 6 (2.7%) to 39 (11.7%) in those two lists.

**Table 2.** Characteristics of assemblages of reef fishes at different locations in the Greater Caribbean region. Percentages of ecotypes in the entire regional fauna, the entire faunas from each local area, and within each of two depth subgroups refer to number of species as a % of the entire fauna and of each depth subgroup. Assemblages include those at Statia in 2017 and 2020 (Statia17 and Statia20), in the Saba EEZ in 2017 (Saba17), of species in the Saba17 fauna that are not currently known to occur at Statia (Saba17-Statia20), of the Saba EEZ in 2020 (Statia20 + Saba17), and of Alligator reef in 2020 (Alligator20). Small species are those with ≤ 10 cm maximum total length. Percentage values for individual sites that are greater than the regional value are shown in red, those below the regional value are in blue.

	**Region**	**Statia20**	**Statia17**	**Saba17**	**Saba20**	**Alligator20**	**Saba17 – Statia20**
**ALL SPECIES (n)**	**903**	**306**	**220**	**377**	**427**	**427**	**121**
Demersal species%	35.0	55.1	66.8	47.5	46.1	49.4	19.0
Benthic species%	65.0	43.1	33.2	52.5	53.9	50.6	81.0
Cryptobenthic species%	59.2	40.8	30.9	49.1	50.1	46.4	73.6
Small cryptobenthic species%	41.6	24.8	15.5	30.2	31.9	24.8	49.6
Core CRF species%	45.8	28.8	20.5	33.4	35.1	27.6	48.8
**SHALLOW SPECIES**%	**85.1**	**87.3**	**97.3**	**93.4**	**88.0**	**95.3**	**90.1**
Non-cryptic species%	40.8	59.6	68.1	50.3	47.6	58.6	23.9
Cryptobenthic species%	59.2	40.4	31.3	49.7	52.4	41.1	76.1
Small cryptobenthic species%	41.3	23.2	15.4	30.7	31.9	25.8	53.2
Core CRF species%	46.2	28.5	21.0	34.1	35.4	29.0	52.3
**DEEP SPECIES**%	**14.9**	**12.7**	**2.7**	**6.6**	**12.0**	**4.7**	**9.9**
Non-cryptic species%	40.3	56.5	83.3	60.0	54.9	75	50.0
Cryptobenthic species%	59.7	43.5	16.7	40.0	45.1	25	50.0
Small cryptobenthic species%	43.3	35.6	16.7	24.0	31.4	5	16.7
Core CRF species%	44.0	38.5	0	24.0	33.3	0	16.7

**Notes**: see methods for classification of ecotypes. For lists of species in the Saba EEZ and Statia2020, and their ecotypic classifications see Suppl. material 2: Table S1. *Pteroisvolitans* and *Pristipomoidesspp* are excluded from Suppl. material 2: Table S1 and the calculations in Table 2. The former is non-native and the specific identity of *Pristipomoides* at Statia is uncertain.

### Ecology – Reef-associated bony fishes

The Statia20 fauna of such species is 38.3% larger than the Statia17 fauna, with numbers of shallow species increasing by 24.8% (from 214 to 267) and of deep species increasing 6.2-fold (from 6 to 39). This led to substantial increases in the relative abundance of deep-reef species, and of benthic, cryptobenthic, small cryptobenthic and core CRFs on both shallow and deep reefs. The Saba17 fauna was 71% larger than that of Statia17, with greater percentages of deep-reef, benthic, cryptobenthic, small cryptobenthic and Core CRFs. The Saba17 fauna was 23% larger than the Statia20 fauna and had a greater proportion of shallow species and fewer deep species, and higher proportions of shallow members of cryptobenthic, small cryptobenthic and Core CRF groups. Thirty-two percent of the Saba17 species were not in the Statia20 fauna. Those 121 species comprised mainly shallow cryptobenthic types, including small-cryptobenthic and Core-CRF species. When those are combined with the Statia20 fauna the resultant Saba20 fauna has substantial increases in the proportions of shallow cryptobenthic, small cryptobenthic and core CRF species compared to the Statia20 fauna. Relative to the regional fauna, however, the faunas of Statia17, Statia20, Saba17, and Saba20 all had deficits of deep species of all types and of shallow cryptobenthic species, including small- and Core-CRF species. The Alligator20 fauna of reef-associated species is the same size as the Saba20 fauna. It has the same characteristics as the Statia17 and Saba17 faunas: a large deficit of deep-reef fishes and deficits of shallow cryptobenthic species, including small- and Core-CRF species. Although there has been some collecting at Alligator reef of shallow cryptobenthic species there has been no submersible-based collecting there.

## Discussion

The efforts of van Kuijk et al. (2015) and Davies and Piontek (2017) substantially increased our knowledge of the known ichthyofauna of Statia, from 215 to 304 species. The information added through the research in 2017 and 2020 has produced a further significant increase, by 33.6%, to 406 species. While the size of the Statia17 fauna was similar to that known for other islands in the Caribbean (Williams et al. 2010; Davies and Piontek 2017) the Statia20 fauna is distinctly larger. That can be attributed to the combination of research on deep-reef fishes by DROP in 2017 and on shallow species by CJE and AME in 2020. Williams et al. (2010) compared the size of the Saba Bank fauna to the faunas of various Caribbean sites and two in the Florida Keys. The size of the large known fauna at one of those Florida sites, Alligator Reef, has increased by ~ 20% since the Williams et al. (2010) study (see Starck et al. 2017; Estapé et al. 2020). However, the current state of knowledge for the other Caribbean sites referred to by Davies and Piontek (2017) and Williams et al. (2010) is unclear.

Zoogeographically the two largest groups of species in the Statia20 fauna are Greater Caribbean endemics and western Atlantic endemics, and the smallest group is of species found in the Indo-Pacific as well as the Atlantic. This mixture is fairly representative of the Greater Caribbean fish fauna as a whole (Robertson and Cramer 2014), and similar to that of nearby St. Croix (Smith-Vaniz and Jelks 2014). The vast majority of the species in the Statia20 fauna are widely distributed in the Greater Caribbean. Among the very few (2.25%) with restricted ranges most information on range-size is available for the shallow species, which belong to two of the most speciose Core CRF families in the Greater Caribbean, the Gobiidae and Chaenopsidae. High levels of local endemism is a feature of some CRF taxa (Brandl et al. 2018) and regionally those two families have substantial proportions of species with restricted ranges, as defined here: 78.7% of 47 chaenopsids and 42.4% of 139 reef-associated gobies (see species maps in Robertson and Van Tassell 2019).

Most species recorded in the Statia17 fauna are readily visible reef fishes, demersal and non-cryptic benthic species commonly found on wider Caribbean reefs, and the proportions of cryptobenthic (particularly small ones) and deep-reef species were relatively low. Davies and Piontek (2017) recognized that both those groups were probably underrepresented in their checklist due to inadequate sampling. Aspects of data collection that affect the adequacy of sampling at a location include its spatial distribution, techniques used, and the depth of sampled habitats. Of all research efforts to date at Statia only the shallow BRUV sampling by van Kuijk et al. (2015) can be regarded as spatially representative, as it was well dispersed around both exposed and sheltered sides of the island. SCUBA-based sampling by Davies and Piontek (2017) and both DROP and the Estapés was largely limited to the more sheltered platform on the western side of the island, and the submersible sampling by DROP was restricted to one small area at the southwest corner of the island shelf. Hence, there are large areas of habitat on the seaward platform and on deep reefs around three quarters of the island that remain unsampled. Furthermore, roving SCUBA surveys are largely limited to providing information on larger, more readily visible demersal and pelagic species (Ackerman and Bellwood 2000; Smith-Vaniz et al. 2006; Alzate et al. 2014). BRUVs are similarly limited: only 10.3% of the 106 species recorded by van Kuijk et al. (2015) are cryptobenthic forms, and only 2.8% are small cryptobenthic species (see Suppl. materials 2, 3: Tables S1, S2).

Rotenone is an ichthyocide commonly used in small quantities by researchers to extract cryptobenthic fishes hiding within reef structures or buried in soft bottoms, and is an important tool for elucidating the contribution of such species to reef-fish faunal assessments (Ackerman and Bellwood 2000; Smith-Vaniz et al. 2006; Robertson and Smith-Vaniz 2008). Davies and Piontek (2017) indicated that sampling using ichthyocides to extract cryptobenthic species hiding within the matrix of the reef at Statia likely would increase the size of the fauna. Rotenone sampling has been employed on shallow reefs of Saba Bank by Williams et al. (2010), and can account for the large numbers of small cryptobenthic species encountered there that are not on the Statia20 checklist: 60% of the 142 species collected by Williams et al. (2010) at Saba bank using that ichthyocide are cryptobenthic forms. Given that that bank is very close to Statia (the two shallow platforms are < 20 km apart) and, since it lacks mangroves, seagrasses and intertidal habitats, the bank may have even lower habitat diversity than Statia. Hence, it seems quite likely that many of the cryptobenthic species, particularly the small ones, found on that bank will be encountered at Statia when appropriate sampling has been done. However, the increase in numbers of shallow cryptobenthic species at Statia from 2017 to 2020 does show that organized searching by skilled citizen scientists can contribute substantially to knowledge of cryptobenthic species. The activities of CJE and AME added 33 shallow cryptobenthic species to the checklist, 31% of the total and 85% of the new records for that ecogroup in the 2020 fauna, and equivalent to 49% of the number present in the Statia17 fauna (Tables 1, 2).

The DROP submersible-based sampling is the only organized research on deep-reef fishes conducted to date at Statia or in the Saba EEZ. It produced more than half the new records in the Statia 2020 fauna, including records of eight recently discovered species that currently lack scientific names. It dramatically increased the numerical and proportional abundance of deep-reef species in the general fauna and in the reef-associated component. A lack of such research at Saba bank and Alligator Reef accounts for the very low abundance of deep-reef fishes at those sites.

The proportional abundances of shallow cryptobenthic species, including small species and core CRFs, are also distinctly lower in the Statia20 fauna than the regional fauna. Even if all 121 reef-associated species in the Saba EEZ that are not known from Statia are assumed to be at Statia those proportions still remain below the regional levels. Some of that difference is probably due to sampling artifacts. However, the proportional abundances of those ecotypes in a local fauna like that of Statia, or Alligator Reef, may always be lower than the regional level. In the Greater Caribbean small cryptobenthic species, particularly Core-CRF species such as blennioids and gobies, often have small geographical ranges (see above), which are scattered in different parts of the region (see Robertson and Van Tassell 2019). While the regional level of the proportional abundance of such taxa is based on an aggregate of many such species from a large area, only a subset of species in those taxa will be found at any single site and their proportional contribution to local faunal richness most likely will be lower than the regional level. The Statia20 fauna includes 33.9% of the Greater Caribbean’s reef-associated fish fauna. That percentage rises to 47.3% in Saba20. Whether a tiny island with a small area of a limited range of habitats is likely to support many more species, and whether pelagic recruitment of reef fishes from nearby islands found around three sides of Statia helps sustain the Statia fauna are both debatable issues that bear on the size of its marine fish fauna.

## Conclusions

The research reported in the present study substantially increased our knowledge of the size of the marine fish fauna of Statia and resulted in the discovery of a significant number of undescribed deep-reef species. Although that island fauna is now one of the best documented in the Greater Caribbean there is still much to do to provide a thorough assessment of its diversity. Collecting with ichthyocide (or anesthetics) is essential for effective sampling of the fauna of small, shallow cryptobenthic reef fishes present there, and sampling of both deep and shallow reef fishes needs to be more effectively distributed across the range of habitats present at the island. No single site in the Caribbean Sea has been subject to sufficiently thorough sampling to provide a clear understanding of the size of its entire marine fish fauna, the size of its reef-associated fish fauna, or even the size of its shallow, reef-associated fauna, let alone its deep-reef fish fauna.

### Permits

Collecting by DROP was performed under Saba/Statia BES Permit No. 120317 to the Foundation Curacao Deep Reef Research Centre.

### Animal-Care Permission

DROP collecting was approved by a Smithsonian Institution Animal Care and Use Committee, approval No. 2014-13 to CCB.

## Supplementary materials

Supplementary material 1 Figure S1 Data type Map of EEZ Explanation note Map of Saba EEZ. https://binary.pensoft.net/file/492394This dataset is made available under the Open Database License (http://opendatacommons.org/licenses/odbl/1.0/). The Open Database License (ODbL) is a license agreement intended to allow users to freely share, modify, and use this Dataset while maintaining this same freedom for others, provided that the original source and author(s) are credited.David Ross Robertson, Carlos J. Estapé, Allison M. Estapé, Ernesto Peña, Luke Tornabene, Carole C. Baldwin

Supplementary material 2 Table S1 Data type Dive site list Explanation note List of dive sites with dates and georeferenced coordinates. https://binary.pensoft.net/file/492395This dataset is made available under the Open Database License (http://opendatacommons.org/licenses/odbl/1.0/). The Open Database License (ODbL) is a license agreement intended to allow users to freely share, modify, and use this Dataset while maintaining this same freedom for others, provided that the original source and author(s) are credited.David Ross Robertson, Carlos J. Estapé, Allison M. Estapé, Ernesto Peña, Luke Tornabene, Carole C. Baldwin

Supplementary material 3 Table S2 Data type Occurences Explanation note Fish species occurrences at Saba and Statia. https://binary.pensoft.net/file/492396This dataset is made available under the Open Database License (http://opendatacommons.org/licenses/odbl/1.0/). The Open Database License (ODbL) is a license agreement intended to allow users to freely share, modify, and use this Dataset while maintaining this same freedom for others, provided that the original source and author(s) are credited.David Ross Robertson, Carlos J. Estapé, Allison M. Estapé, Ernesto Peña, Luke Tornabene, Carole C. Baldwin

Supplementary material 4 Plate S1 Data type Photographs Explanation note Voucher photographs of fishes. https://binary.pensoft.net/file/492397This dataset is made available under the Open Database License (http://opendatacommons.org/licenses/odbl/1.0/). The Open Database License (ODbL) is a license agreement intended to allow users to freely share, modify, and use this Dataset while maintaining this same freedom for others, provided that the original source and author(s) are credited.David Ross Robertson, Carlos J. Estapé, Allison M. Estapé, Ernesto Peña, Luke Tornabene, Carole C. Baldwin

Supplementary material 5 Plate S2 Data type Photographs Explanation note Voucher photographs of fishes. https://binary.pensoft.net/file/492398This dataset is made available under the Open Database License (http://opendatacommons.org/licenses/odbl/1.0/). The Open Database License (ODbL) is a license agreement intended to allow users to freely share, modify, and use this Dataset while maintaining this same freedom for others, provided that the original source and author(s) are credited.David Ross Robertson, Carlos J. Estapé, Allison M. Estapé, Ernesto Peña, Luke Tornabene, Carole C. Baldwin

Supplementary material 6 Plate S3 Data type Photographs Explanation note Voucher photographs of fishes. https://binary.pensoft.net/file/492399This dataset is made available under the Open Database License (http://opendatacommons.org/licenses/odbl/1.0/). The Open Database License (ODbL) is a license agreement intended to allow users to freely share, modify, and use this Dataset while maintaining this same freedom for others, provided that the original source and author(s) are credited.David Ross Robertson, Carlos J. Estapé, Allison M. Estapé, Ernesto Peña, Luke Tornabene, Carole C. Baldwin

Supplementary material 7 Plate S4 Data type Photographs Explanation note Voucher photographs of fishes. https://binary.pensoft.net/file/492400This dataset is made available under the Open Database License (http://opendatacommons.org/licenses/odbl/1.0/). The Open Database License (ODbL) is a license agreement intended to allow users to freely share, modify, and use this Dataset while maintaining this same freedom for others, provided that the original source and author(s) are credited.David Ross Robertson, Carlos J. Estapé, Allison M. Estapé, Ernesto Peña, Luke Tornabene, Carole C. Baldwin

Supplementary material 8 Plate S5 Data type Photographs Explanation note Voucher photographs of fishes. https://binary.pensoft.net/file/492401This dataset is made available under the Open Database License (http://opendatacommons.org/licenses/odbl/1.0/). The Open Database License (ODbL) is a license agreement intended to allow users to freely share, modify, and use this Dataset while maintaining this same freedom for others, provided that the original source and author(s) are credited.David Ross Robertson, Carlos J. Estapé, Allison M. Estapé, Ernesto Peña, Luke Tornabene, Carole C. Baldwin

Supplementary material 9 Plate S6 Data type Photographs Explanation note Voucher photographs of fishes. https://binary.pensoft.net/file/492402This dataset is made available under the Open Database License (http://opendatacommons.org/licenses/odbl/1.0/). The Open Database License (ODbL) is a license agreement intended to allow users to freely share, modify, and use this Dataset while maintaining this same freedom for others, provided that the original source and author(s) are credited.David Ross Robertson, Carlos J. Estapé, Allison M. Estapé, Ernesto Peña, Luke Tornabene, Carole C. Baldwin

Supplementary material 10 Plate S7 Data type Photographs Explanation note Voucher photographs of fishes. https://binary.pensoft.net/file/492403This dataset is made available under the Open Database License (http://opendatacommons.org/licenses/odbl/1.0/). The Open Database License (ODbL) is a license agreement intended to allow users to freely share, modify, and use this Dataset while maintaining this same freedom for others, provided that the original source and author(s) are credited.David Ross Robertson, Carlos J. Estapé, Allison M. Estapé, Ernesto Peña, Luke Tornabene, Carole C. Baldwin

Supplementary material 11 Plate S8 Data type Photographs Explanation note Voucher photographs of fishes. https://binary.pensoft.net/file/492404This dataset is made available under the Open Database License (http://opendatacommons.org/licenses/odbl/1.0/). The Open Database License (ODbL) is a license agreement intended to allow users to freely share, modify, and use this Dataset while maintaining this same freedom for others, provided that the original source and author(s) are credited.David Ross Robertson, Carlos J. Estapé, Allison M. Estapé, Ernesto Peña, Luke Tornabene, Carole C. Baldwin

Supplementary material 12 Plate S9 Data type Photographs Explanation note Voucher photographs of fishes. https://binary.pensoft.net/file/492405This dataset is made available under the Open Database License (http://opendatacommons.org/licenses/odbl/1.0/). The Open Database License (ODbL) is a license agreement intended to allow users to freely share, modify, and use this Dataset while maintaining this same freedom for others, provided that the original source and author(s) are credited.David Ross Robertson, Carlos J. Estapé, Allison M. Estapé, Ernesto Peña, Luke Tornabene, Carole C. Baldwin

Supplementary material 13 Plate S10 Data type Photographs Explanation note Voucher photographs of fishes. https://binary.pensoft.net/file/492406This dataset is made available under the Open Database License (http://opendatacommons.org/licenses/odbl/1.0/). The Open Database License (ODbL) is a license agreement intended to allow users to freely share, modify, and use this Dataset while maintaining this same freedom for others, provided that the original source and author(s) are credited.David Ross Robertson, Carlos J. Estapé, Allison M. Estapé, Ernesto Peña, Luke Tornabene, Carole C. Baldwin

Supplementary material 14 Plate S11 Data type Photographs Explanation note Voucher photographs of fishes. https://binary.pensoft.net/file/492407This dataset is made available under the Open Database License (http://opendatacommons.org/licenses/odbl/1.0/). The Open Database License (ODbL) is a license agreement intended to allow users to freely share, modify, and use this Dataset while maintaining this same freedom for others, provided that the original source and author(s) are credited.David Ross Robertson, Carlos J. Estapé, Allison M. Estapé, Ernesto Peña, Luke Tornabene, Carole C. Baldwin

Supplementary material 15 Plate S12 Data type Photographs Explanation note Voucher photographs of fishes. https://binary.pensoft.net/file/492408This dataset is made available under the Open Database License (http://opendatacommons.org/licenses/odbl/1.0/). The Open Database License (ODbL) is a license agreement intended to allow users to freely share, modify, and use this Dataset while maintaining this same freedom for others, provided that the original source and author(s) are credited.David Ross Robertson, Carlos J. Estapé, Allison M. Estapé, Ernesto Peña, Luke Tornabene, Carole C. Baldwin

Supplementary material 16 Plate S13 Data type Photographs Explanation note Voucher photographs of fishes. https://binary.pensoft.net/file/492409This dataset is made available under the Open Database License (http://opendatacommons.org/licenses/odbl/1.0/). The Open Database License (ODbL) is a license agreement intended to allow users to freely share, modify, and use this Dataset while maintaining this same freedom for others, provided that the original source and author(s) are credited.David Ross Robertson, Carlos J. Estapé, Allison M. Estapé, Ernesto Peña, Luke Tornabene, Carole C. Baldwin
