# Supplementary material for: The marine fishes of St Eustatius Island, northeastern Caribbean: an annotated, photographic catalog
Source: Zookeys. 2020 Dec 30;1007:145–80. doi: 10.3897/zookeys.1007.58515 (PMC7788074; doi:10.3897/zookeys.1007.58515)

**Supplemental Figure S1:** Map of the Exclusive Economic Zone of Saba. That zone, outlined by the dashed line, includes Saba, Sint Eustatius, and the southern half of Sint Maarten island. Source: Figure 2 of Hoetjes and Carpenter (2010).


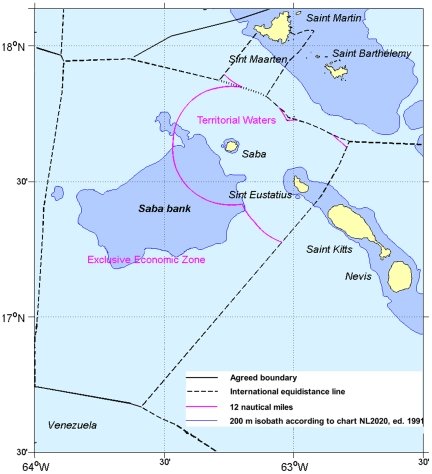

Supplement: Supplementary material 1 — Figure S1 [file zookeys-1007-145-s001.docx]
