# Supplementary figures and images for: The marine fishes of St Eustatius Island, northeastern Caribbean: an annotated, photographic catalog
Source: Zookeys. 2020 Dec 30;1007:145–80. doi: 10.3897/zookeys.1007.58515 (PMC7788074; doi:10.3897/zookeys.1007.58515)

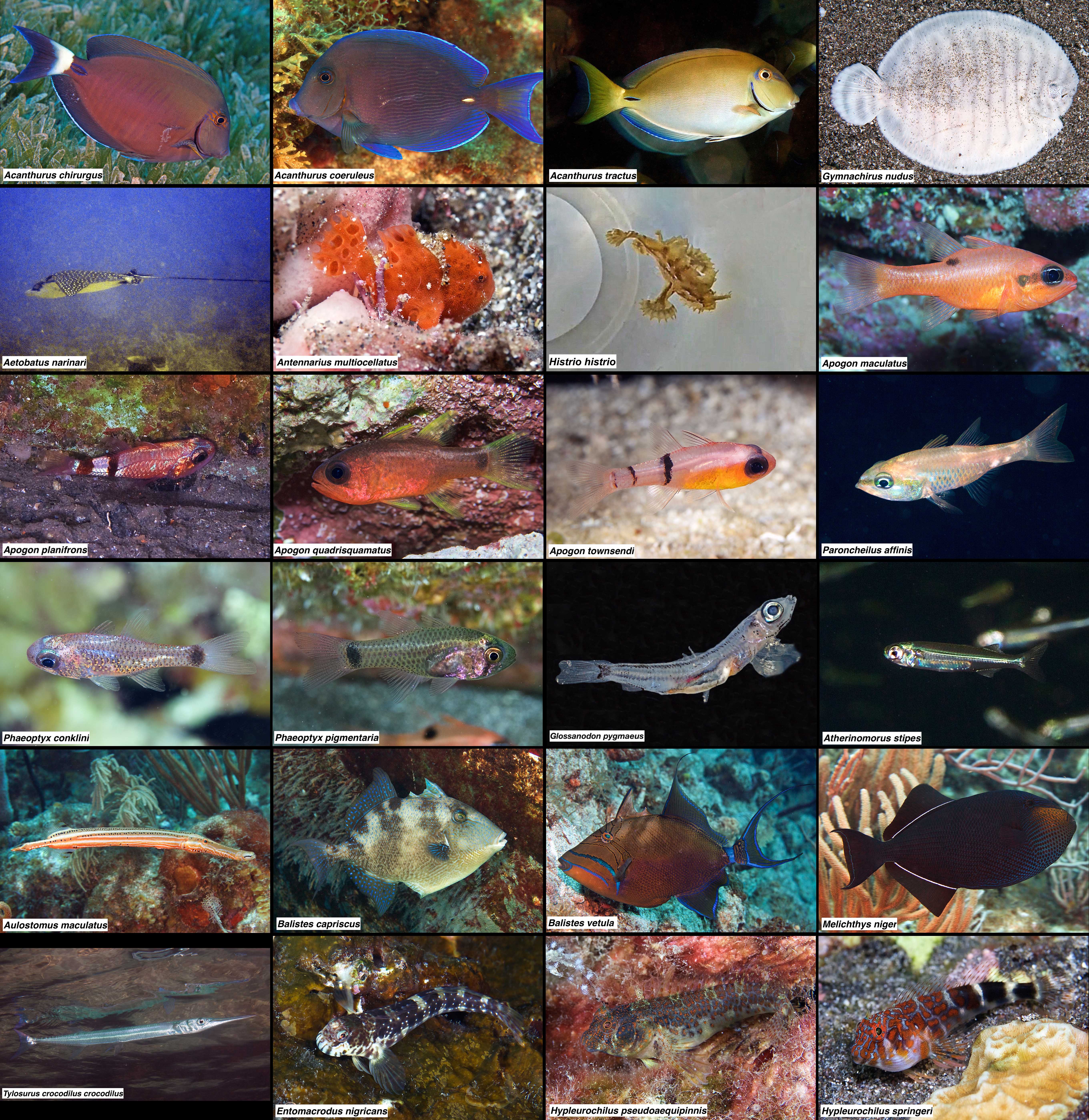

Supplement: Supplementary material 4 — Plate S1 [file zookeys-1007-145-s004.jpg]

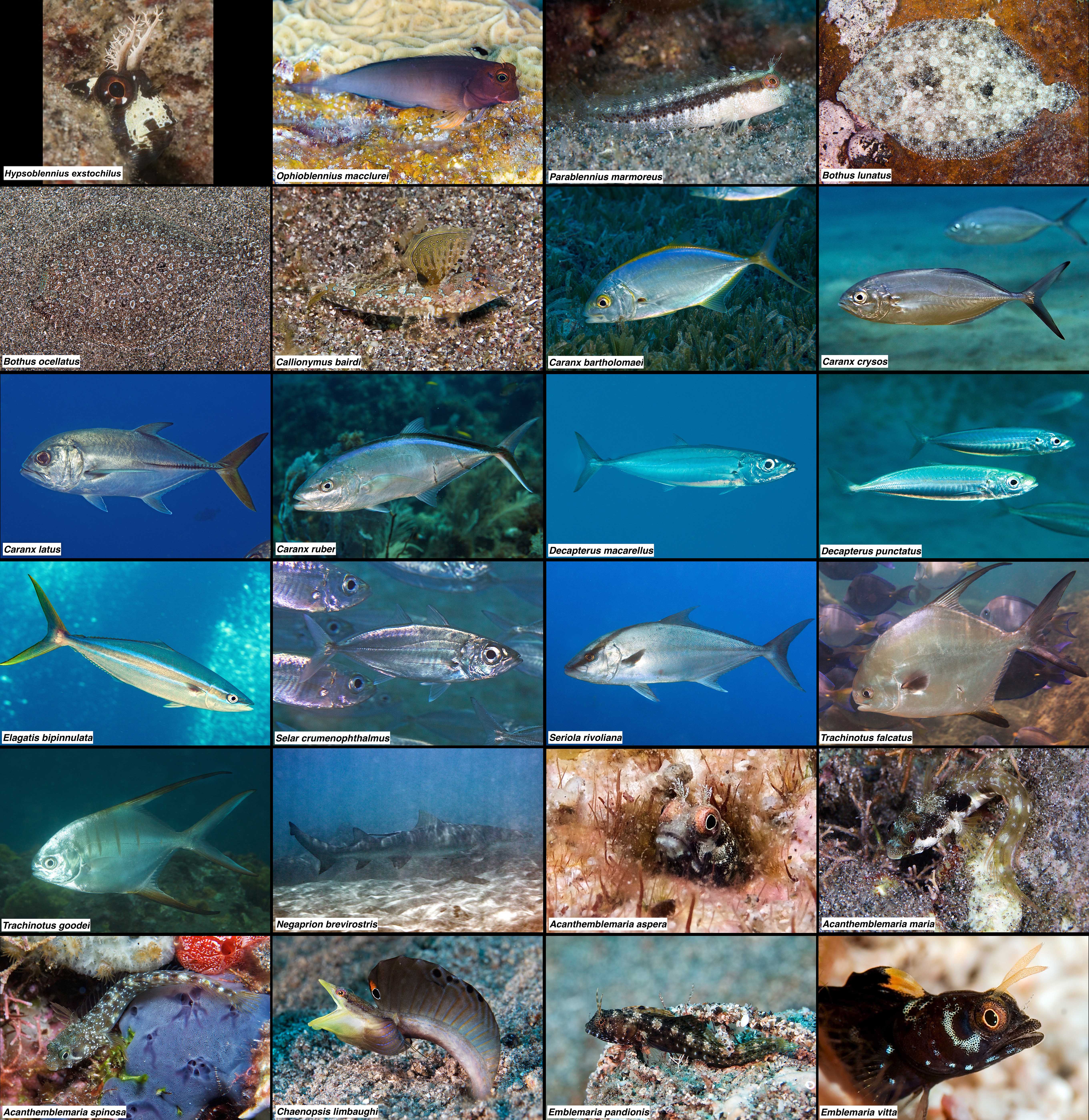

Supplement: Supplementary material 5 — Plate S2 [file zookeys-1007-145-s005.jpg]

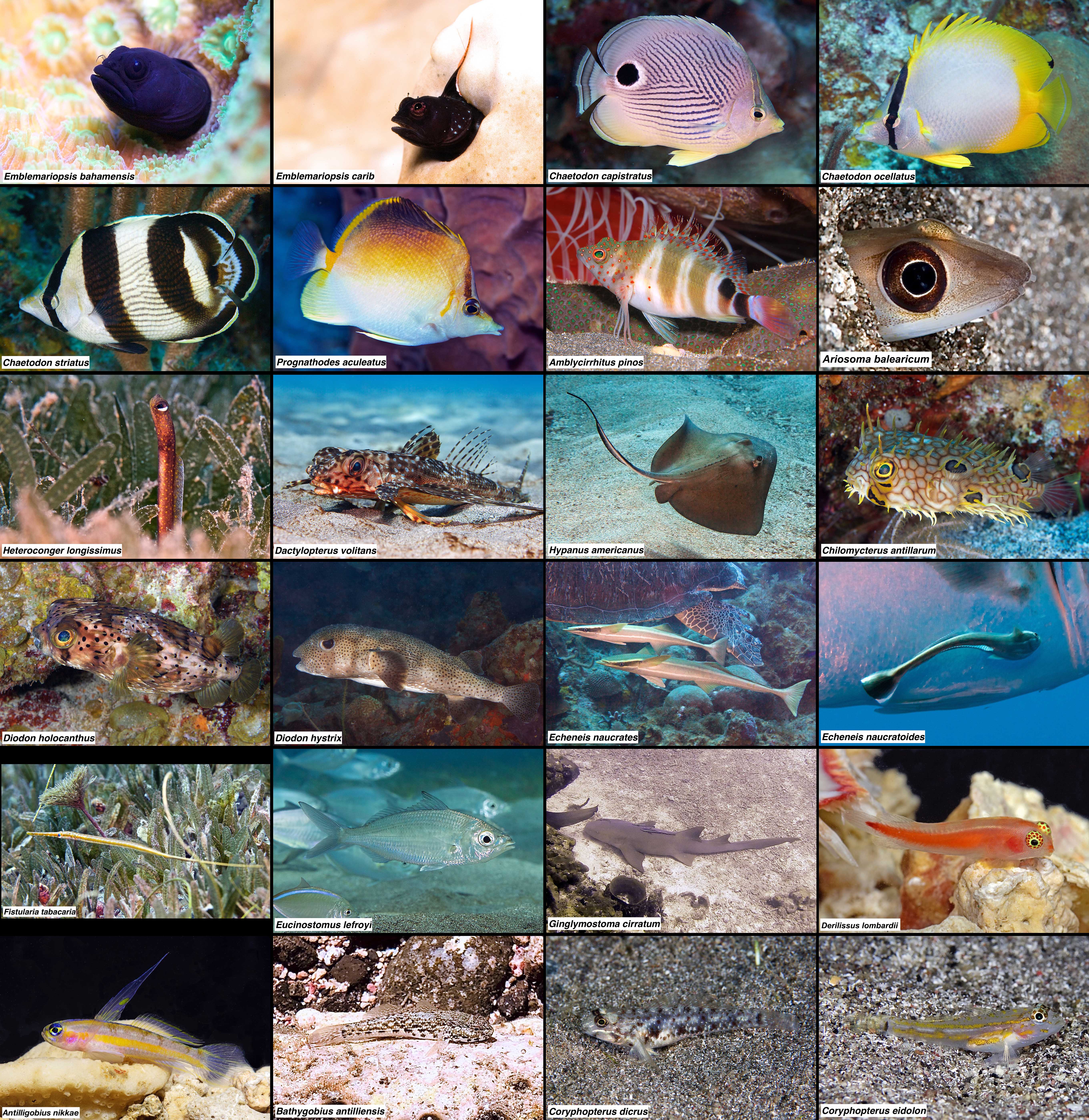

Supplement: Supplementary material 6 — Plate S3 [file zookeys-1007-145-s006.jpg]

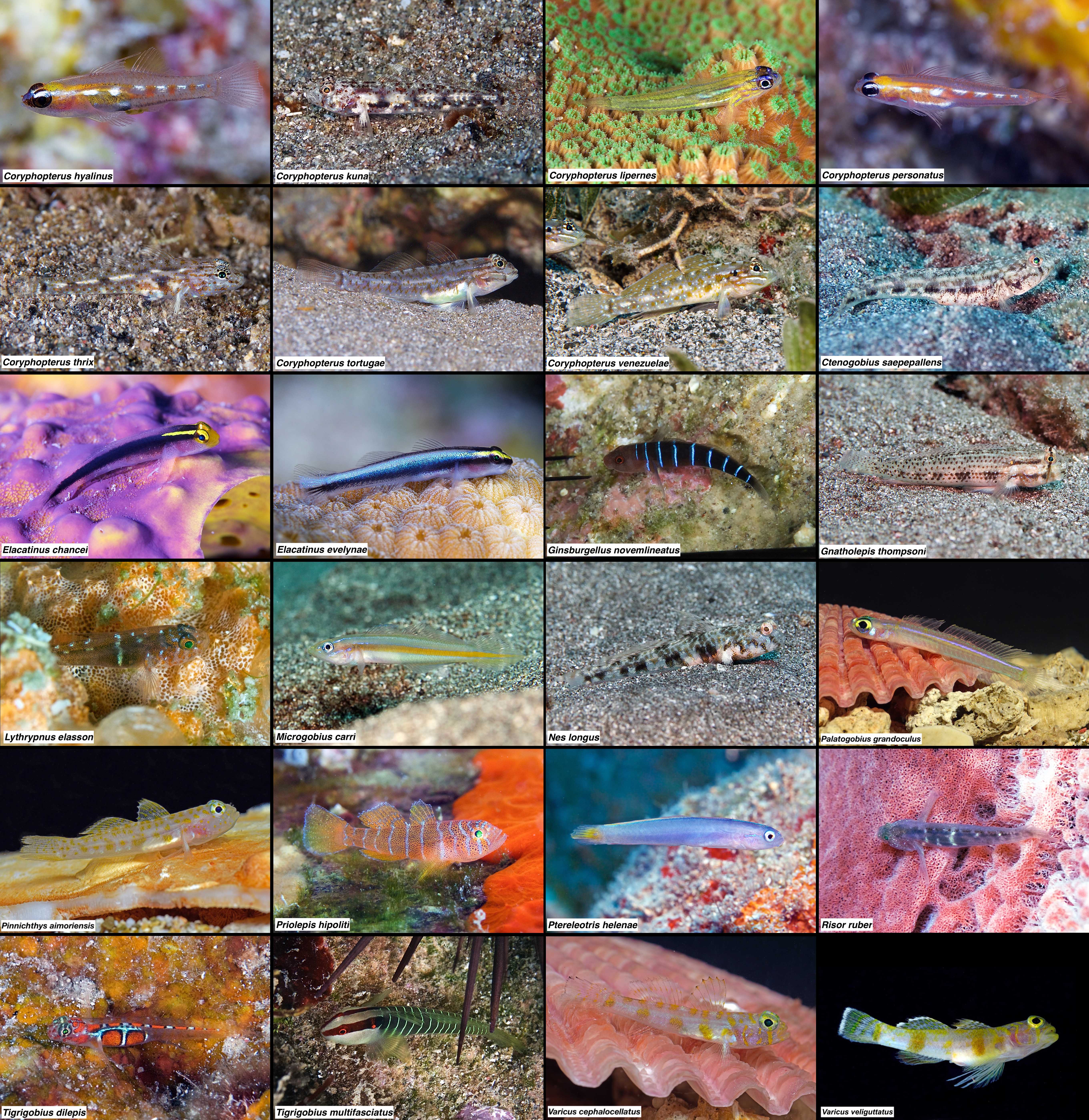

Supplement: Supplementary material 7 — Plate S4 [file zookeys-1007-145-s007.jpg]

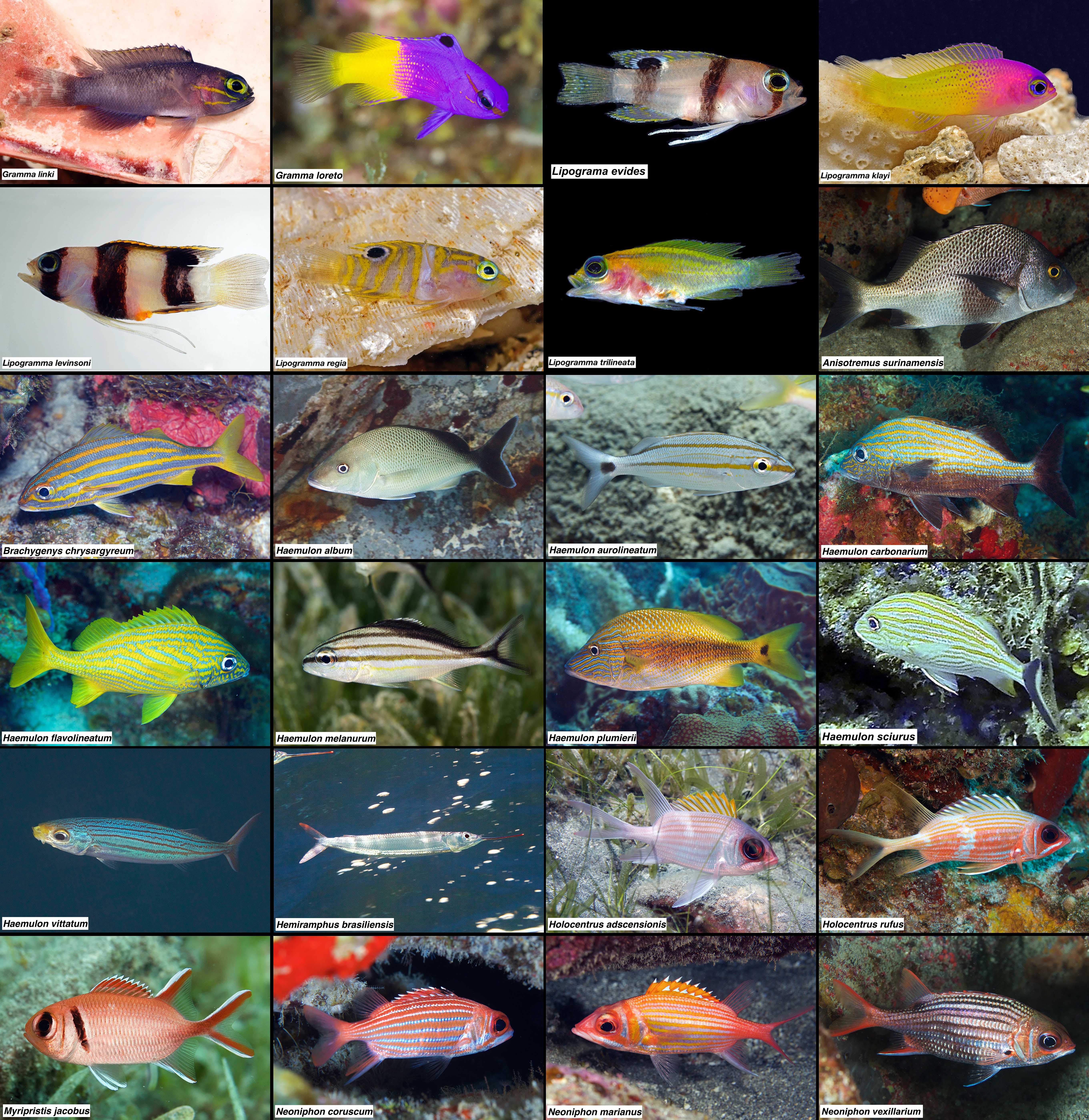

Supplement: Supplementary material 8 — Plate S5 [file zookeys-1007-145-s008.jpg]

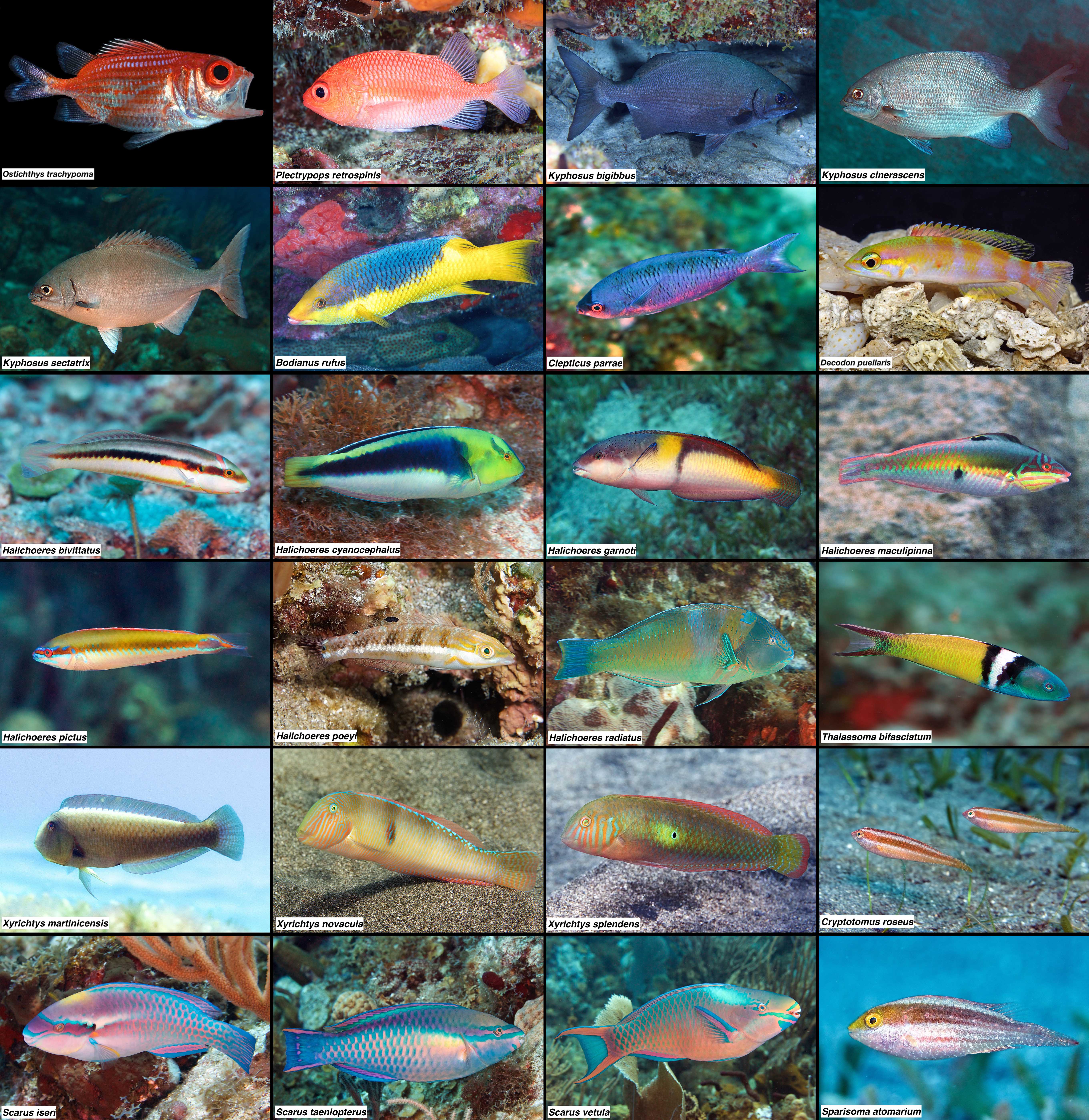

Supplement: Supplementary material 9 — Plate S6 [file zookeys-1007-145-s009.jpg]

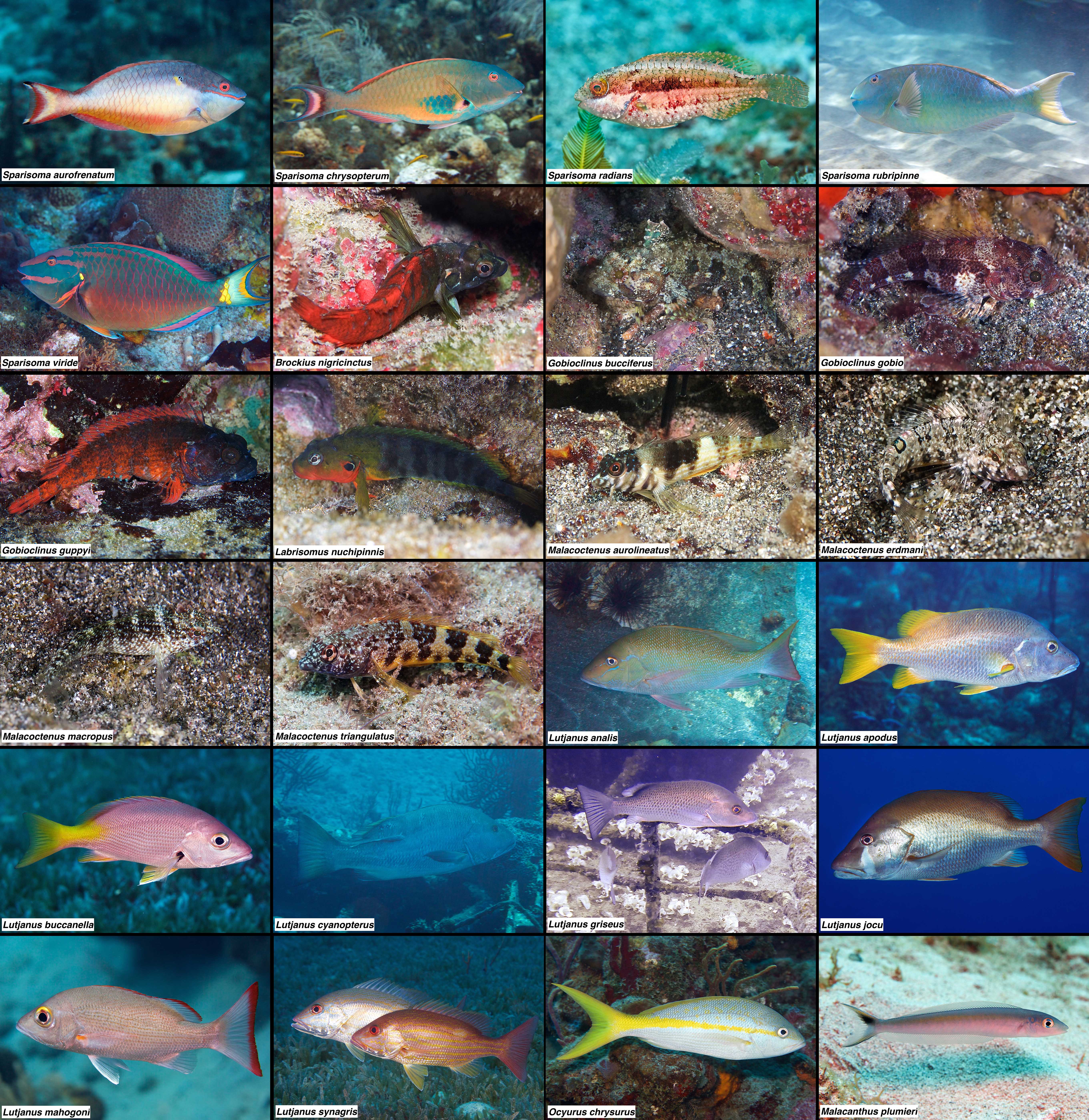

Supplement: Supplementary material 10 — Plate S7 [file zookeys-1007-145-s010.jpg]

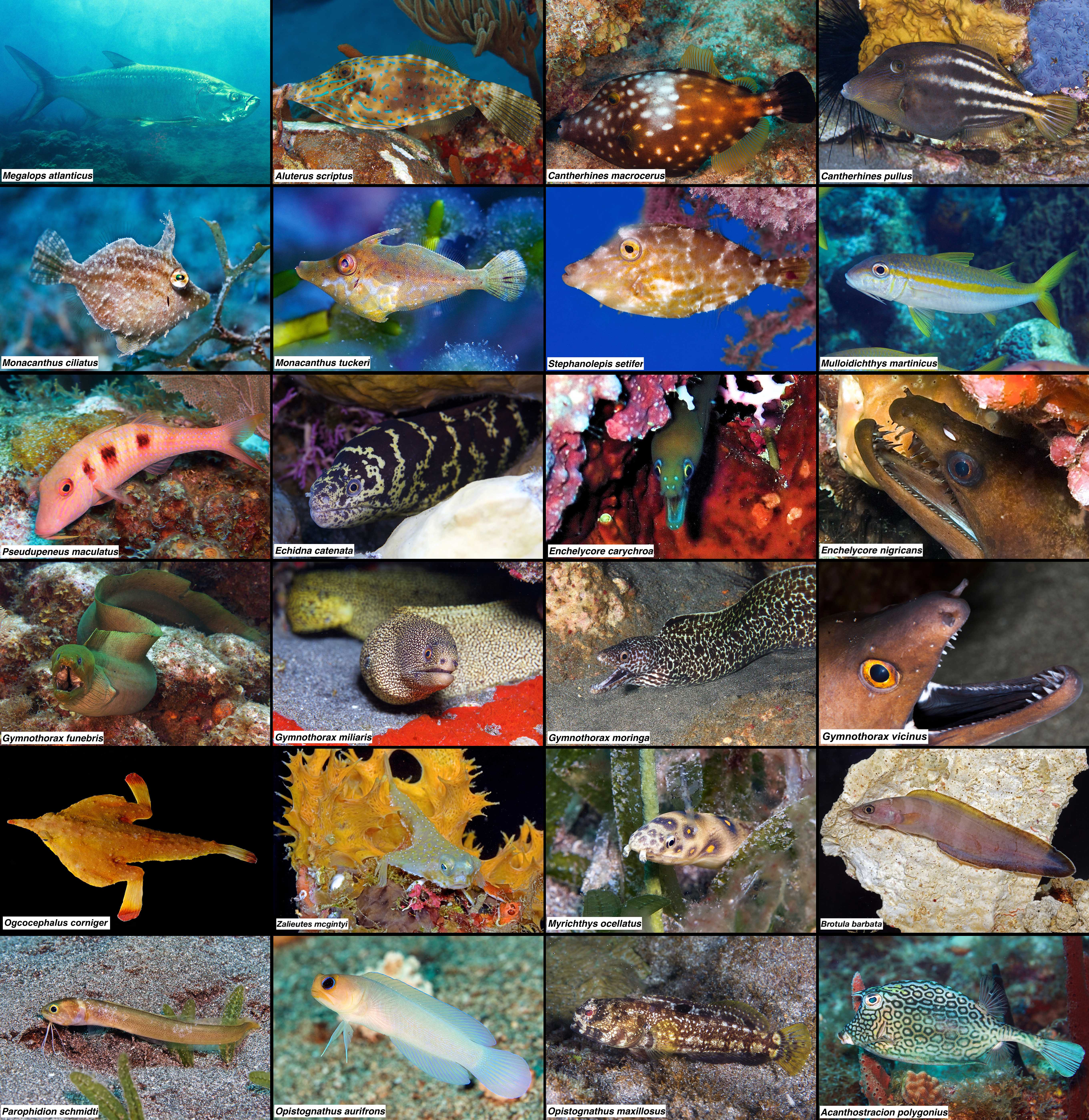

Supplement: Supplementary material 11 — Plate S8 [file zookeys-1007-145-s011.jpg]

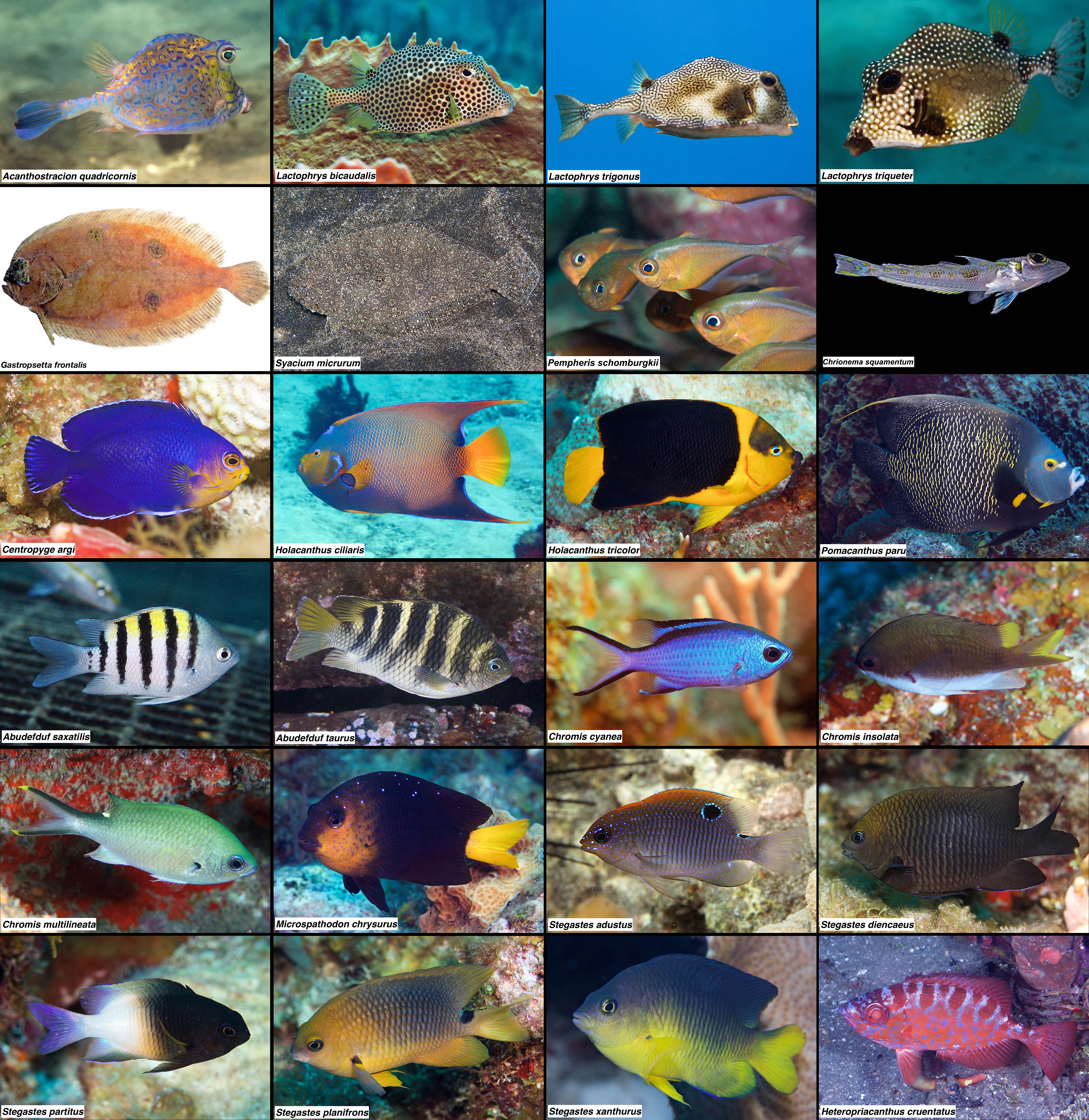

Supplement: Supplementary material 12 — Plate S9 [file zookeys-1007-145-s012.jpg]

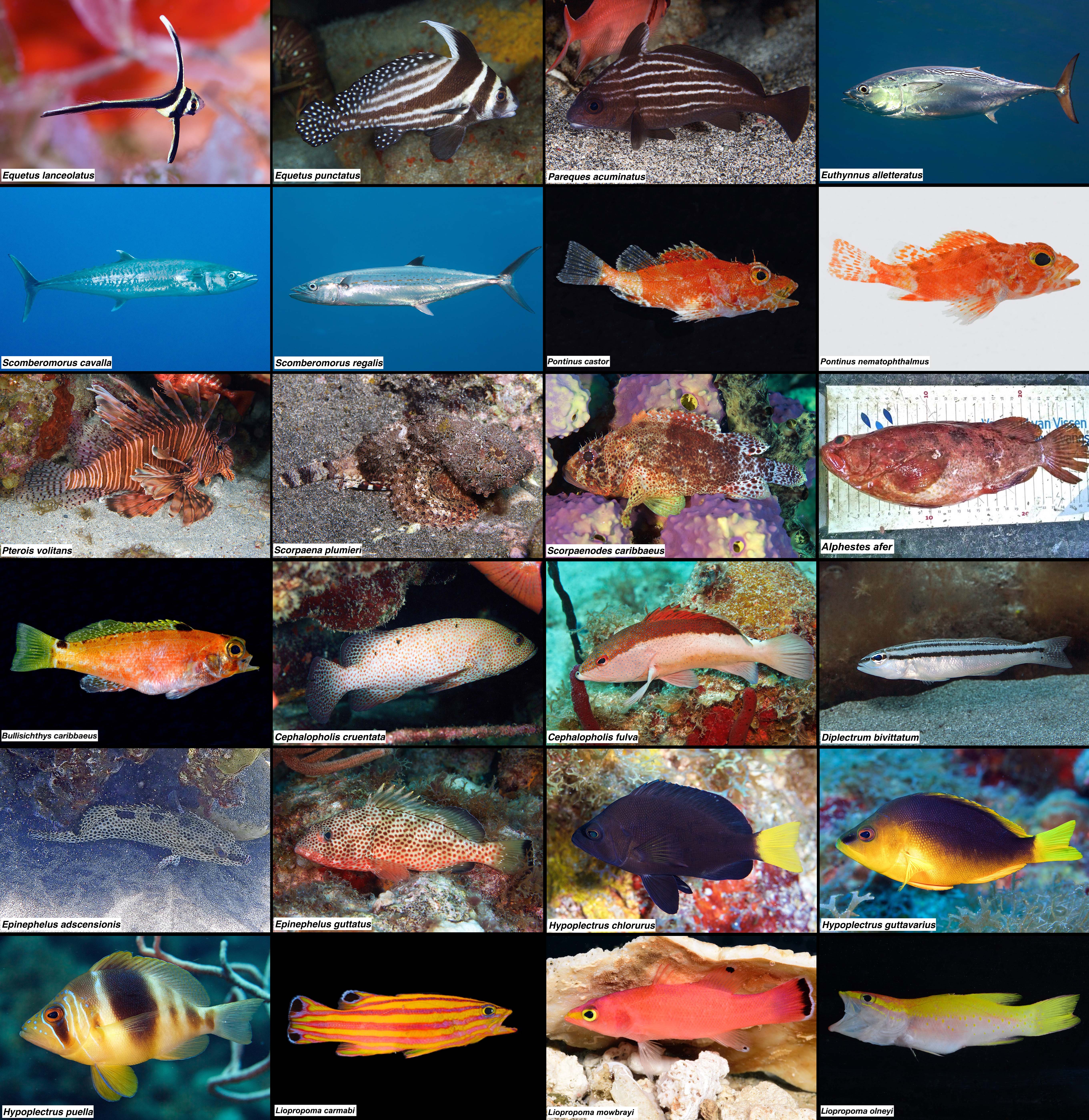

Supplement: Supplementary material 13 — Plate S10 [file zookeys-1007-145-s013.jpg]

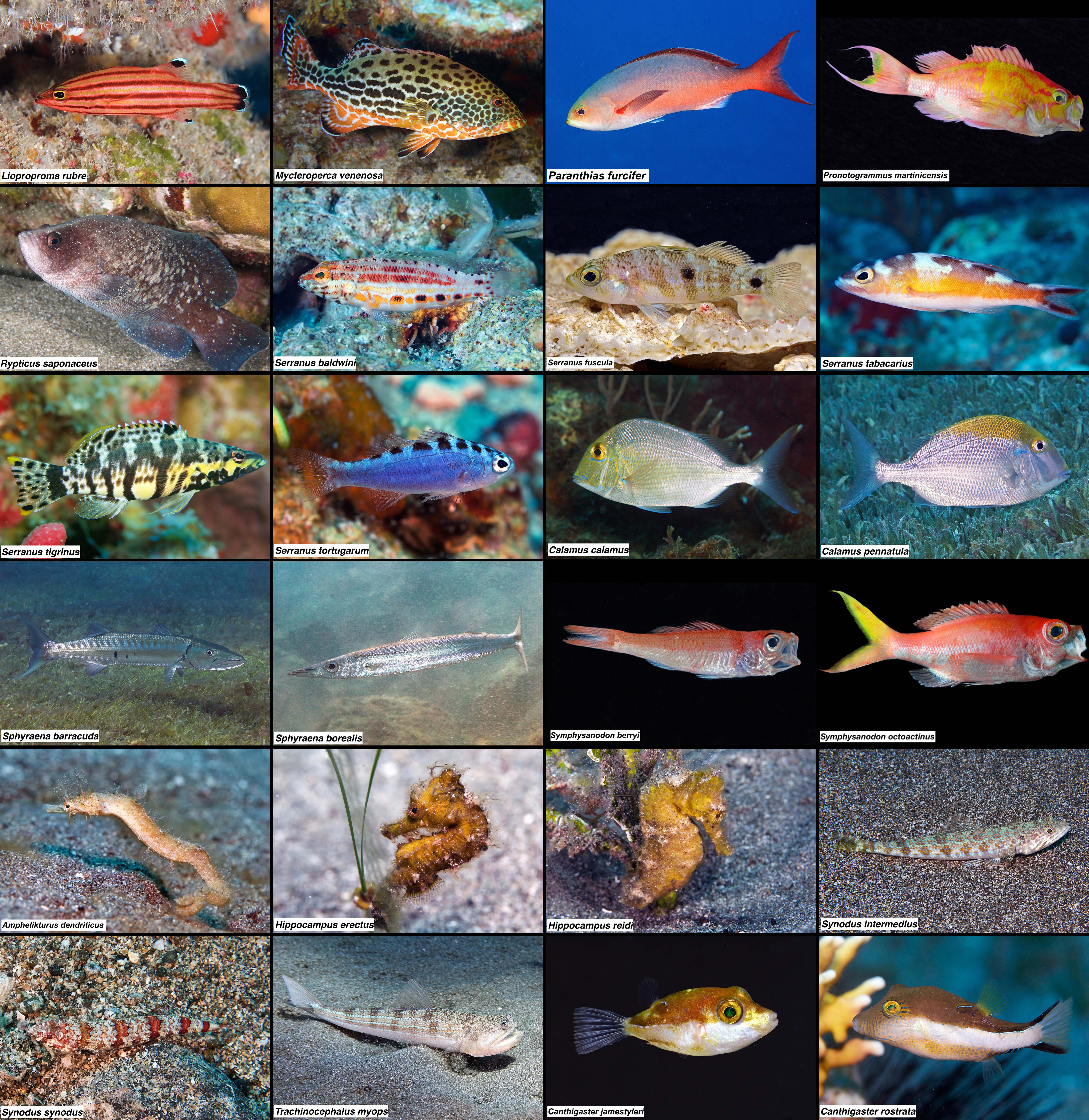

Supplement: Supplementary material 14 — Plate S11 [file zookeys-1007-145-s014.jpg]

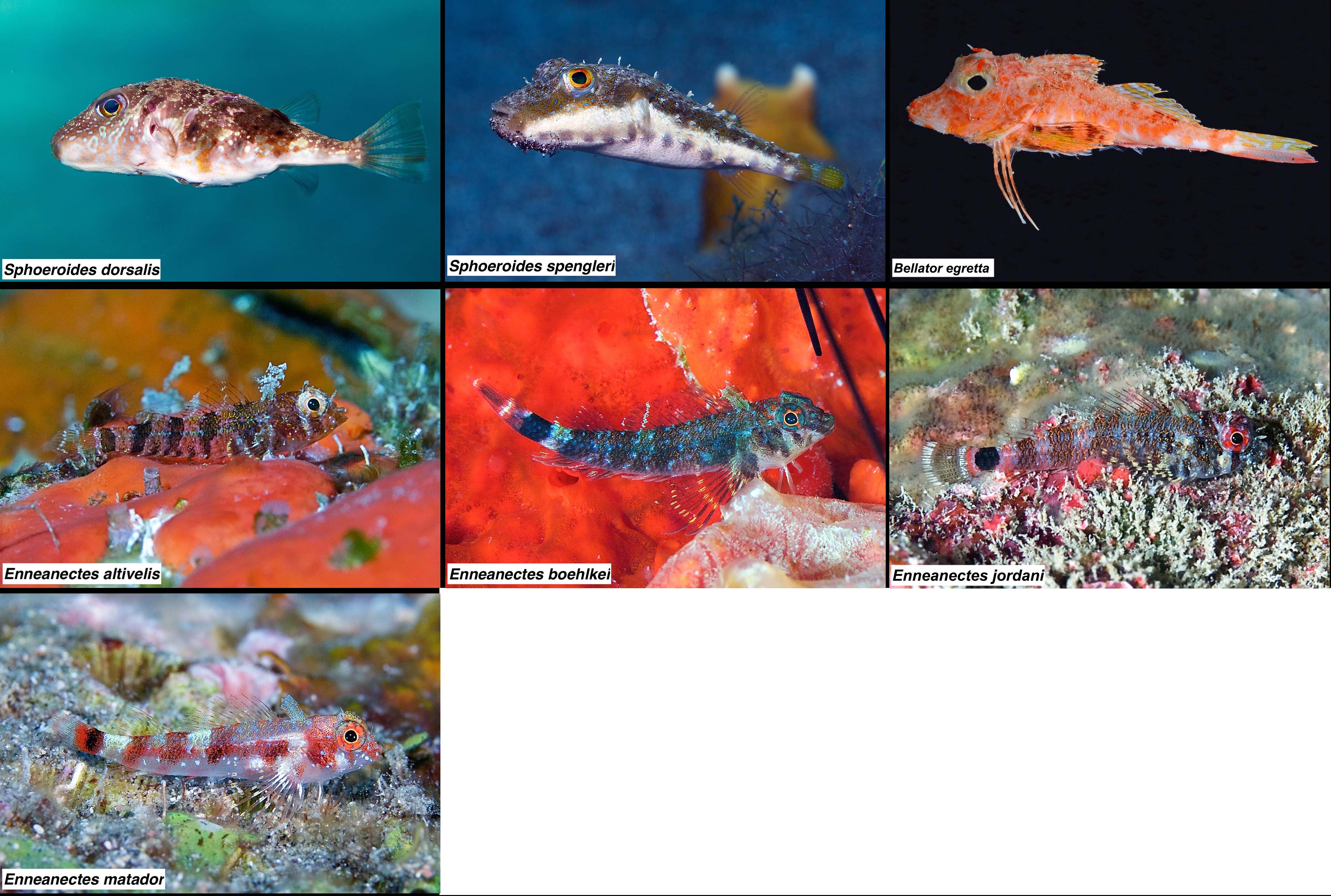

Supplement: Supplementary material 15 — Plate S12 [file zookeys-1007-145-s015.jpg]

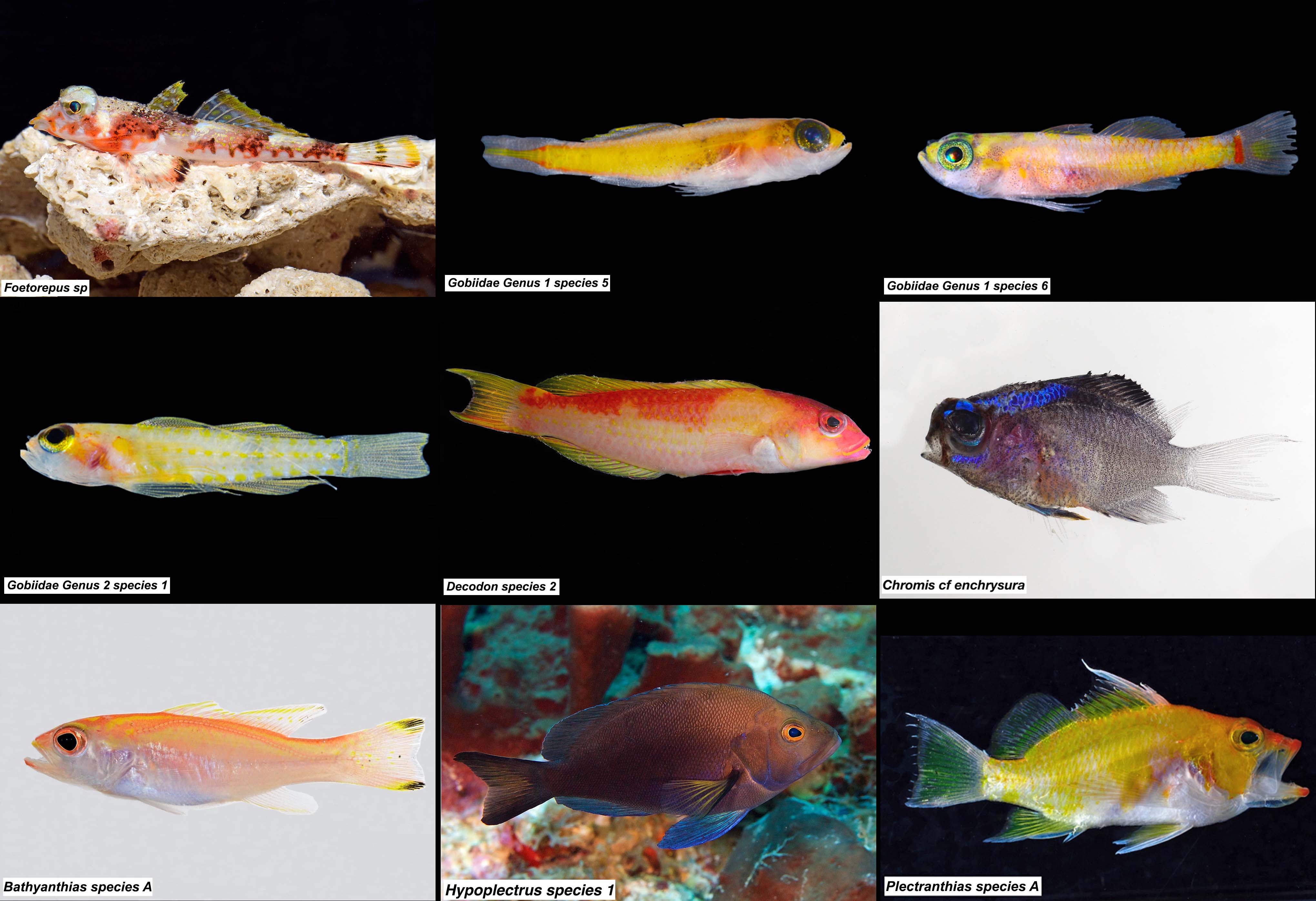

Supplement: Supplementary material 16 — Plate S13 [file zookeys-1007-145-s016.jpg]
